# Tubule-derived CCN1 drives renal repair via α_v_β_5_-STAT6-ARG1-dependent reprogramming of macrophages

**DOI:** 10.1038/s41419-025-08340-2

**Published:** 2025-12-21

**Authors:** Ningxin Zhang, Chenyu Li, Yanlu Xin, Zhuo Song, Tianyang Li, Ruizhe Zhao, Minghao Gu, Lingyu Xu, Yanfei Wang, Xiaofei Man, Lin Che, Hang Liu, Chen Guan, Yan Xu

**Affiliations:** 1 Department of Nephrology, the Affiliated Hospital of Qingdao University, Qingdao, Shandong China; 2 Department of Medicine, Renal Electrolyte and Hypertension Division, Perelman School of Medicine, University of Pennsylvania, Philadelphia, PA USA

**Keywords:** Acute kidney injury, Monocytes and macrophages, Growth factor signalling, Cell death and immune response, Cell growth

## Abstract

Macrophages play a critical role in injury and repair following acute kidney injury (AKI), but their regulatory mechanisms remain incompletely understood. Cellular communication network factor 1 (CCN1), a secreted matricellular protein and early biomarker of AKI, may regulate macrophage function during kidney injury. In this study, we first investigated CCN1’s interaction with macrophages in a murine model of ischemia-reperfusion (I/R)-induced AKI. The role of CCN1 was further investigated using recombinant protein administration, a renal tubular epithelial cell (RTEC)-specific CCN1 knockdown mouse model via adeno-associated virus, and in vitro studies with bone marrow-derived macrophages (BMDMs). We found that in response to injury, RTECs upregulated and secreted CCN1, which colocalized with infiltrating F4/80^+^ macrophages. RTEC-specific CCN1 knockdown exacerbated renal injury and reduced macrophage infiltration, as confirmed by H&E, KIM-1 and F4/80 staining. Transcriptomic profiling of kidney tissues revealed that CCN1 expression was associated with immune cell infiltration, particularly macrophages, while RNA-seq analysis of BMDMs demonstrated that CCN1 promoted pro-repair arginase1 *(Arg1)*^+^ macrophage differentiation and activated the STAT6 signaling pathway. BayesPrism deconvolution further confirmed the enrichment of *Arg1*^hi^ macrophages following CCN1 treatment. Co-immunoprecipitation coupled with mass spectrometry identified integrin α_v_β_5_ as a direct CCN1-binding partner mediating STAT6/ARG1 activation. Functionally, CCN1-treated macrophages enhanced RTECs' proliferation in vitro and in vivo, an effect abolished by ARG1 inhibition or macrophage depletion. In conclusion, CCN1 regulates macrophages via the α_v_β_5_-STAT6-ARG1 axis to promote tubular epithelial proliferation and improve kidney function in I/R-AKI, highlighting a novel tubular-immune communication pathway and potential therapeutic targets for ischemic AKI.

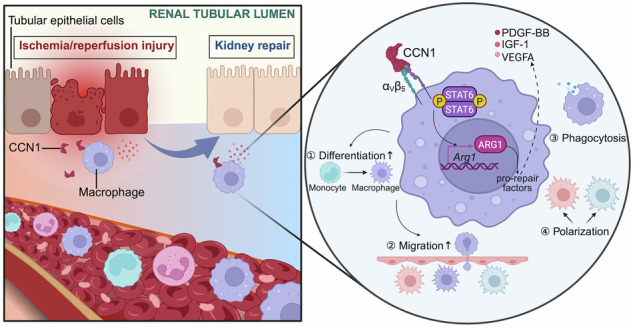

## Introduction

Acute kidney injury (AKI) is characterized by a sudden loss of renal function, manifested as a decline in glomerular filtration rate (GFR), reduced urine output, and elevated serum creatinine levels. AKI affects 10–15% of all hospitalized patients and over 50% of those in intensive care units, with more than 2 million deaths globally each year attributed to AKI [1, 2]. As a dynamic process, the extent of recovery after AKI depends on the regeneration and functional restoration of injured renal epithelial cells. Incomplete or maladaptive repair following AKI can lead to secondary chronic kidney disease (CKD) [3, 4]. Among the various etiologies, ischemia-reperfusion (I/R) injury is one of the most common causes of AKI [5, 6]. Understanding the molecular mechanisms governing ischemic AKI progression and recovery is, therefore, critical for developing targeted therapies to improve clinical outcomes.

A growing body of evidence has shown that macrophages are important regulators of the renal response to injury [7, 8]. During AKI, renal tubular epithelial cells (RTECs) release damage-induced signals, including chemokines and damage-associated molecular patterns (DAMPs), which mediate macrophage recruitment and activation [9]. Increasing evidence demonstrates that specific macrophage subsets actively contribute to renal repair by promoting RTECs proliferation [10, 11], such as mannose receptor C-type 1 (*Mrc1*)^+^ [12] and arginase1 (*Arg1*)^+^ [13] populations. A recent spatial transcriptome study revealed the dynamic changes of macrophage subsets during AKI progression: the “pro-repair Macrophage” increased significantly during the early 3-day period after injury, but their proportion decreased significantly in the chronic stage, suggesting that a decrease in pro-repair macrophages may be associated with poor renal repair after AKI [14]. Therefore, early intervention to increase the proportion of pro-repair macrophages may become a potential strategy to improve AKI outcomes.

Cellular communication network factor 1 (CCN1) is a 42-kDa extracellular matrix protein that plays a pivotal role in cell adhesion, proliferation, migration, differentiation, and angiogenesis under both physiological and pathological conditions [15, 16]. Recent studies have increasingly highlighted CCN1 serves as a master regulator of macrophage function, exhibiting remarkably diverse immunomodulatory effects depending on pathological context. In acute bacterial infections, CCN1 enhances macrophage phagocytosis of bacteria for clearance and acts as a DAMP to activate inflammatory responses [17]. The role of CCN1 in metabolic diseases appears more complex, where in non-alcoholic steatohepatitis it promotes pro-fibrotic macrophage activation [18], while in fatty liver disease it facilitates macrophage migration within metastatic tumor microenvironments [19]. Conversely, in autoimmune myocarditis, CCN1 has been shown to suppress macrophage migration, alleviating inflammation and tissue damage [20]. These seemingly paradoxical effects suggest that CCN1 exerts disease and context-specific regulatory functions on macrophages, underscoring its pivotal but complex role in immune modulation. Our previous work demonstrated that CCN1 is rapidly upregulated in RTECs during the early phase of I/R-AKI and exerts protective effects against injury of various etiologies by mitigating apoptosis [21, 22]. Although associations between CCN1 and RTECs have been established in both experimental models and human biopsy specimens [23, 24], the precise role of CCN1 in regulating macrophage function during kidney injury remains unclear.

In this study, we employed in vitro and in vivo approaches, including RTEC-specific CCN1 knockdown mouse model, recombinant protein administration, RNA-seq with deconvolution analysis by using BayesPrism model, and a macrophage-tubular epithelial cell co-culture system, to investigate the role of CCN1 in macrophage regulation during ischemic AKI. Our findings demonstrate that RTEC-secreted CCN1 promotes pro-repair macrophage reprogramming via the α_v_β_5_-STAT6-ARG1 axis to enhance tubular proliferation and improve renal outcomes.

## Results

### CCN1 is upregulated and secreted by RTECs during I/R-AKI

**Fig. 1:**
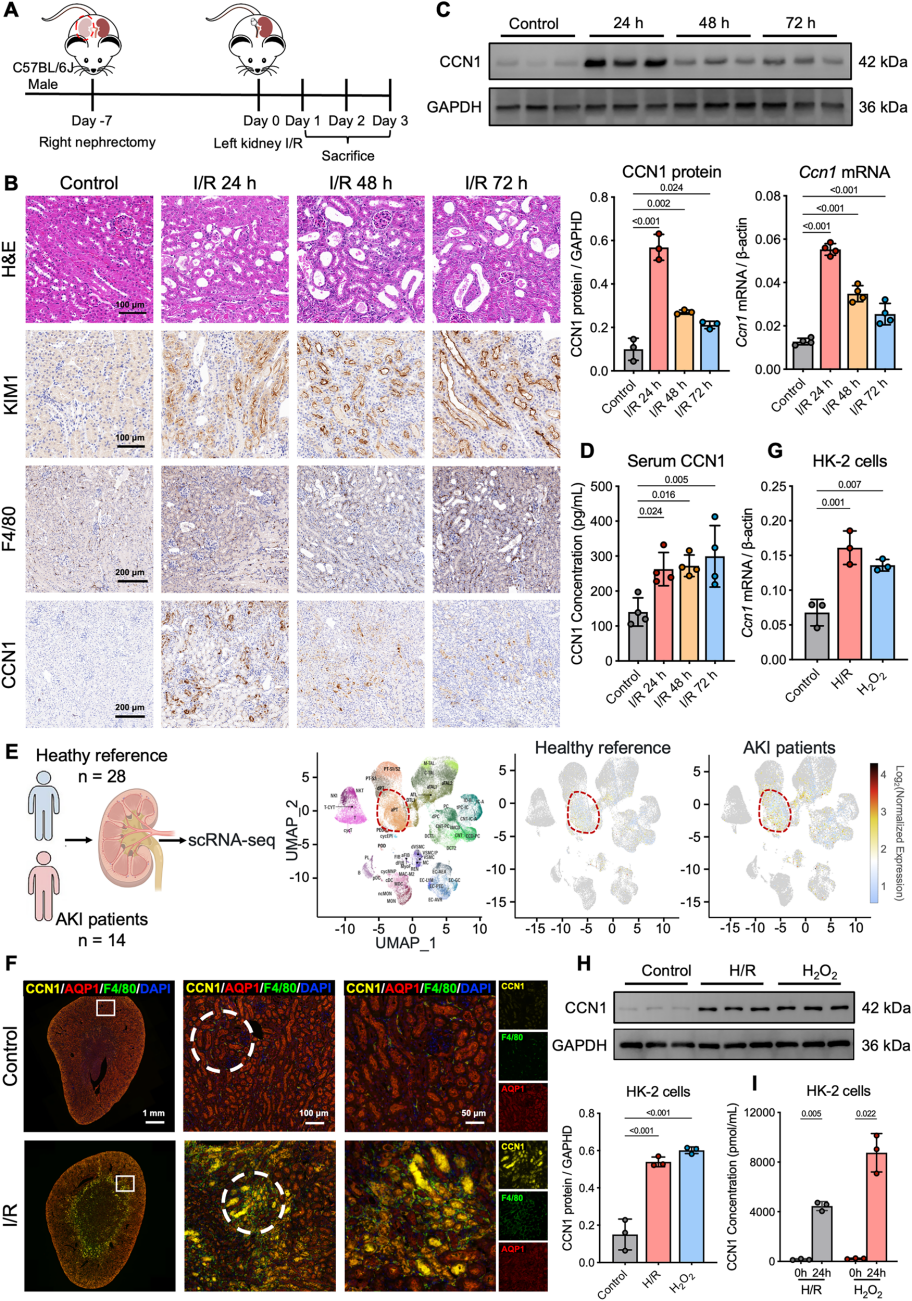
RTECs express and release CCN1 following renal I/R injury. **A** A diagram illustrating the setup of the experiment uxIRI. **B** Representative images of H&E staining, KIM1, F4/80, and CCN1 immunostaining in kidney tissues. **C** Renal CCN1 protein and mRNA expression. **D** Serum CCN1 levels in mice after I/R. *n* = 4 mice per group. **E** CCN1 expression in RTECs from AKI patients by single-cell RNA-seq. **F** Representative fluorescent micrographs and quantification analysis of CCN1 (yellow), AQP1 (red), and F4/80 (green) staining in I/R-AKI mice kidney. *n* = 4 mice per group. **G** CCN1 mRNA expression levels in HK-2 cells after H/R or H_2_O_2_ stimulation. **H** CCN1 protein expression levels in HK-2 cells after H/R or H_2_O_2_ stimulation. **I** Secreted CCN1 protein levels in culture supernatants of HK-2 cells after H/R or H_2_O_2_ stimulation. *n* = 3 per group. Data are presented as mean ± SD.

We first established a murine model of unilateral renal I/R injury with contralateral nephrectomy (uxIRI) to mimic AKI (Fig. 1A). Biochemical analyses revealed significantly elevated blood urea nitrogen and serum creatinine levels at 24 h after I/R, indicating impaired renal function, followed by a gradual decrease at 48 h and 72 h, with the serum creatinine level essentially returning to normal at 72 h (Supplementary Fig. 1A). Histological examination using hematoxylin and eosin (H&E) staining demonstrated classical features of tubular injury after I/R, including tubular epithelial cell swelling, loss of brush border, and tubular dilation (Fig. 1B, Supplementary Fig. 1B). Consistently, expression of kidney injury molecule-1 (KIM1) was markedly upregulated in RTECs post-I/R (Fig. 1B, Supplementary Fig. 1B). Immunostaining for F4/80 indicated a substantial increase in macrophage infiltration in the kidney after I/R, predominantly localized to the corticomedullary junction, with a peak at 72 h (Fig. 1B, Supplementary Fig. 1B). The characteristic time-dependent changes in renal function markers, tubular injury patterns, and macrophage infiltration collectively validated the I/R-AKI model.

Furthermore, immunohistochemical analysis revealed markedly elevated CCN1 expression in I/R-AKI kidneys, predominantly localized to cortical RTECs bordering the medullary region (Fig. 1B). Quantitative assessment showed this upregulation peaked at 24 h after I/R with approximately a sixfold increase and remained elevated at both 48 h and 72 h compared to control mice (Supplementary Fig. 1B). Consistent with immunohistochemical findings, CCN1 protein and mRNA levels in renal tissue were upregulated markedly after I/R, reaching maximal intensity at 24 h post-I/R and maintaining elevated expression through 72 h compared with control group (Fig. 1C). Notably, reflecting its secretory properties, circulating CCN1 levels showed similar increases, with progressive elevation from 24 to 72 h after I/R (Fig. 1D). To evaluate clinical relevance, we analyzed NIH Kidney Precision Medicine Project (KPMP) single-cell RNA-seq data from healthy references (*n* = 28) and AKI patients (*n* = 14) (Fig. 1E). CCN1 expression was significantly elevated in AKI, mainly in RTECs (Fig. 1E), consistent with its upregulation in mouse I/R-induced AKI. These data demonstrated that RTECs upregulate and secrete CCN1 during I/R-induced AKI in mice, with a comparable increase in human AKI. In the kidneys of I/R-AKI mice, CCN1 was primarily localized in AQP1-labeled proximal tubular epithelial cells, with F4/80^+^ macrophages densely infiltrating areas adjacent to CCN1-high tubules (Fig. 1F, Supplementary Fig. 1C). Given the secretory nature of CCN1 and its spatial association with macrophages, we hypothesized that tubule-derived CCN1 might be involved in regulating neighboring macrophage behavior. To verify the expression of CCN1 after tubular epithelial cell injury, we established two injury models in HK-2 cells using hypoxia/reoxygenation (H/R) and H_2_O_2_ stimulation. Both treatments consistently induced significant upregulation of CCN1, with mRNA levels increasing approximately twofold and intracellular protein accumulation rising nearly threefold compared to untreated controls (Fig. 1G, H). Notably, CCN1 protein levels in the culture supernatants were significantly increased to 4000 and 12,000 pg/mL following H/R or H_2_O_2_ exposure, respectively (Fig. 1I). These findings suggest that injured RTECs actively upregulate and secrete CCN1, establishing the prerequisite conditions for potential tubule-macrophage communication during I/R-AKI.

### Exogenous CCN1 administration protects against I/R-AKI by modulating macrophage populations

**Fig. 2:**
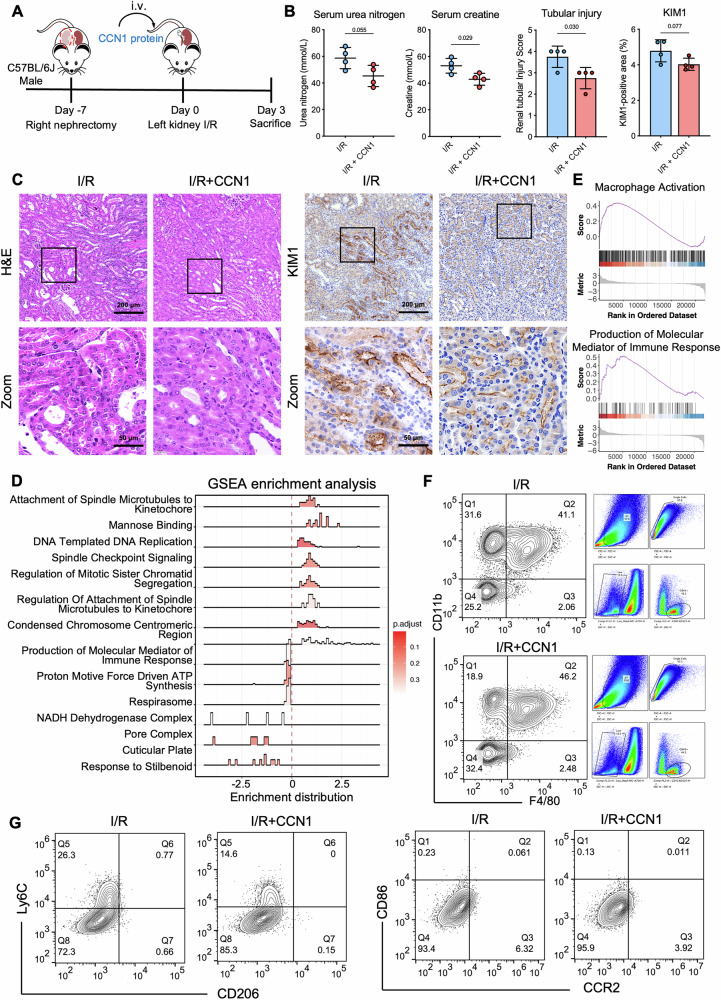
Recombinant CCN1 ameliorates renal injury and modulates macrophages. **A** A diagram illustrating the CCN1 protein administration in mice after I/R. **B** Blood urea nitrogen and serum creatinine levels in mice after I/R. **C** Representative images and quantification analysis of H&E staining, and KIM1 immunostaining in kidney tissues. **D** Density ridge plots showing the expression distributions of core-enriched gene sets identified by GSEA. **E** Selected enrichment plots from GSEA based on the gene enrichment profiles on CCN1-treated I/R kidneys versus I/R controls. **F** Gating strategy for CD45^+^F4/80^+^CD11b^+^ cells in the kidney after I/R by flow cytometry. **G** Subsequent gating for macrophage polarization markers, including CD206, Ly6C, CD86 and CCR2 in the kidney after I/R by flow cytometry. *n* = 4 mice per group. Data are presented as mean ± SD.

To investigate the role of CCN1 in renal injury and its potential function as a secreted protein modulating immune cells, we administered recombinant CCN1 protein to mice via tail vein injection (Fig. 2A). Compared with I/R mice, CCN1-treated mice showed a significant reduction in blood urea nitrogen and creatinine, reduction in kidney pathological injury as shown by H&E staining, and decreased expression of kidney injury marker KIM1 (Fig. 2B, C). To further elucidate the mechanisms underlying the protective effects of CCN1 on kidney injury, we performed RNA sequencing on kidney tissues from treated mice. GSEA of the transcriptome data revealed that CCN1 treatment was associated with immune-related pathways, especially those involved in the production of immune response mediators and macrophage activation (Fig. 2D, E). To validate these findings, we next analyzed kidney immune cell populations by flow cytometry. CCN1 treatment increased macrophages, characterized by elevated CD45^+^CD11b^+^F4/80^+^ cells in the kidney (Fig. 2F). Macrophages exhibit substantial plasticity, allowing them to adapt to environmental cues and participate in both injury and repair phases of stages of I/R-AKI. Traditionally, macrophages are classified as M1 (CD86^+^CCR2^+^), which produce pro-inflammatory cytokines, and M2 (Ly6C^−^CD206^+^), which contribute to tissue repair. Flow cytometry analysis showed that CCN1 did not alter CD86^+^, CCR2^+^ or CD206⁺ populations, but significantly increased Ly6C⁻ cells (Fig. 2G), suggesting a potential modulation of alternative macrophage subsets beyond the traditional M1/M2 paradigm. In contrast, CCN1 treatment did not significantly affect total renal T cells or the CD8^+^ and CD4^+^ subsets (Supplementary Fig. 2). Collectively, these results establish that exogenous CCN1 ameliorates I/R-AKI and modulates Ly6C⁻ macrophage subpopulations.

### RTEC-specific CCN1 knockdown exacerbates AKI and reprograms macrophage responses

**Fig. 3:**
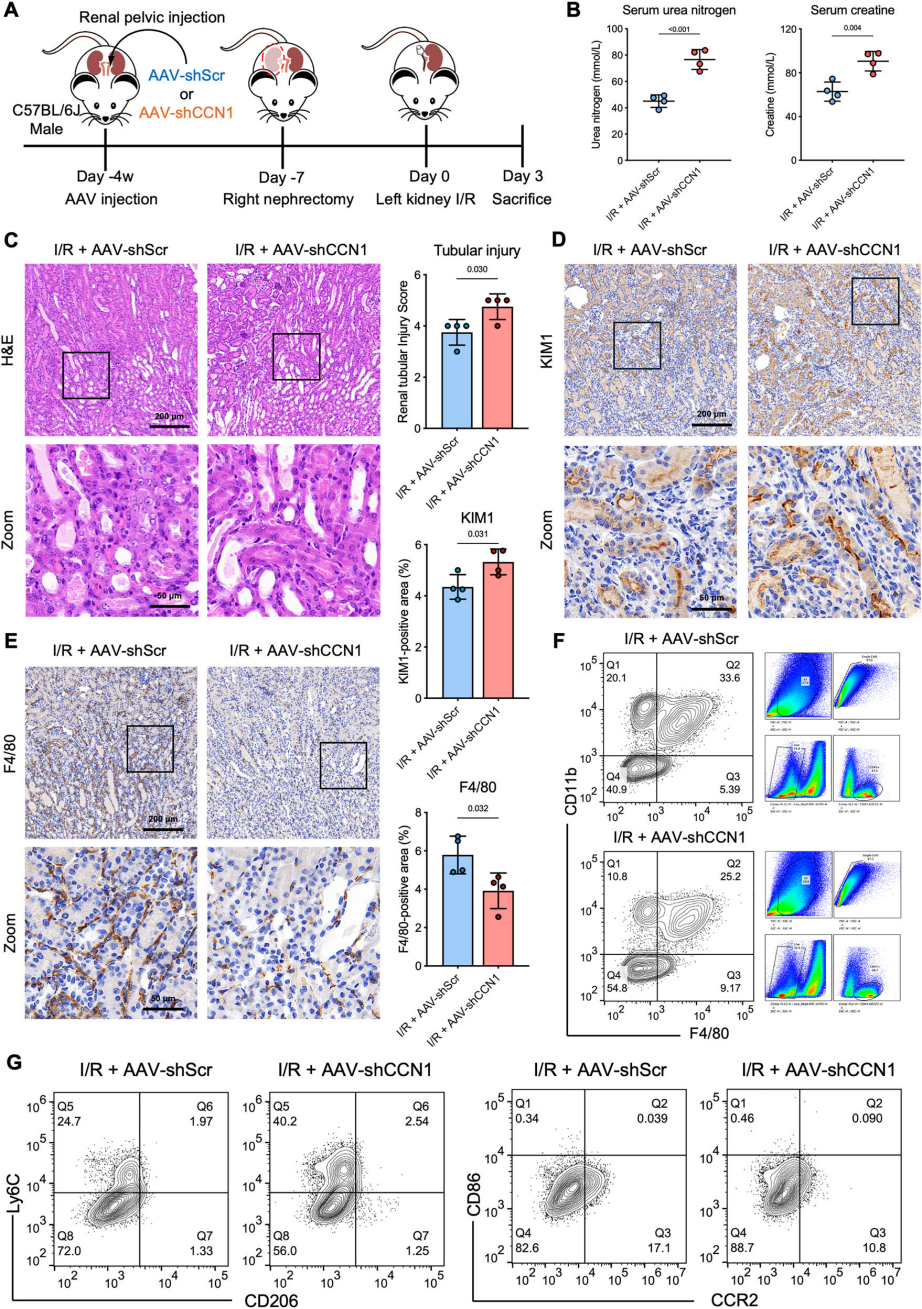
RTEC-specific CCN1 knockdown exacerbates AKI and reduces macrophage infiltration. **A** A diagram of uxIRI setup in a tubule-specific CCN1 knockdown model induced by renal pelvic AAV9-Ksp-shCCN1 injection. **B** Blood urea nitrogen and serum creatinine levels in mice after I/R. Representative images and quantification analysis of **C** H&E staining, **D** KIM1 and **E** F4/80 immunostaining in kidney tissues. **F** Gating strategy for CD45^+^F4/80^+^CD11b^+^ cells in the kidney after I/R by flow cytometry. **G** Subsequent gating for macrophage polarization markers, including CD86, CCR2, Ly6C, and CD206 in the kidney after I/R by flow cytometry. *n* = 4 mice per group. Data are presented as mean ± SD.

To further validate the role of CCN1 in tubular-macrophage communication during I/R-induced AKI, we generated RTEC-specific CCN1 knockdown mice using AAV (Fig. 3A). C57BL/6 J mice received renal pelvic injections of AAV9-Ksp-EGFP-CCN1 shRNA (AAV-shCCN1) or control AAV9-Ksp-EGFP-Scramble (AAV-shScr) (Supplementary Fig. 3A). Four weeks after injection, AAV9-shCCN1 selectively infected RTECs and markedly reduced CCN1 mRNA and protein expression in the kidney cortex, confirming RTEC specificity (Supplementary Fig. 3B, C). Compared with control mice, CCN1-knockdown mice displayed elevated blood urea nitrogen and serum creatinine levels, indicating aggravated renal dysfunction (Fig. 3B). Histological analysis further showed CCN1 knockdown mice had more severe renal damage, as indicated by exacerbated pathological injury and increased KIM1 expression (Fig. 3C, D). Immunohistochemical staining showed that the number of F4/80^+^ macrophages in renal tissues was significantly reduced in kidney tubule-specific CCN1-knockdown mice compared with AAV-scramble controls following I/R injury (Fig. 3E). Flow cytometry demonstrated that CCN1 knockdown significantly reduced CD45^+^CD11b^+^F4/80^+^ cells in kidney (Fig. 3F), with no significant changes in CD86^+^, CCR2^+^ or CD206^+^ subsets, but a marked reduction in Ly6C^-^ macrophages (Fig. 3G). Together, these findings indicate that CCN1 produced by RTECs contributes to renal protection during AKI, at least in part by regulating macrophage responses.

### CCN1 reprograms macrophages toward a reparative phenotype

**Fig. 4:**
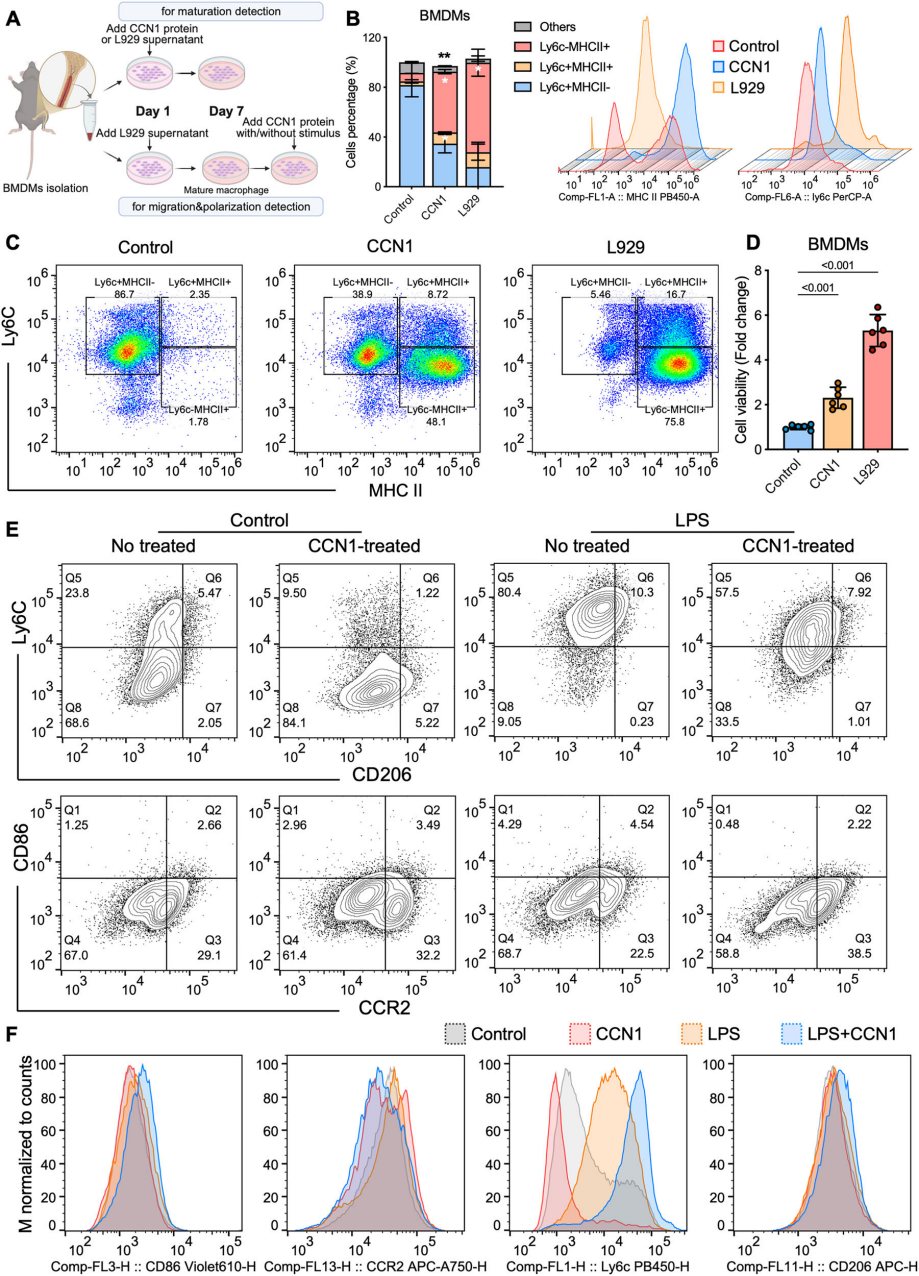
CCN1 promotes macrophage differentiation and polarization. **A** A diagram of BMDMs isolation and CCN1 protein treatment. **B**, **C** Flow cytometry analysis of Ly6C⁺MHCII⁻ monocytes and Ly6C⁻MHCII⁺ macrophages following CCN1 stimulation. *n* = 3 per group. **D** CCK-8 assay of cell viability and adherence after CCN1 treatment. *n* = 6 per group. **E** Contour plot of CD86⁺, CCR2⁺, Ly6C⁻ and CD206⁺ BMDMs by flow cytometry. **F** Histogram of CD86, CCR2, Ly6C and CD206 of BMDMs by flow cytometry. *n* = 3 per group. Data are presented as mean ± SD.

To determine whether tubule-derived CCN1 directly regulates macrophage function, we performed in vitro experiments using bone marrow-derived macrophages (BMDMs) treated with recombinant CCN1 protein (Fig. 4A). During progressive differentiation, bone marrow progenitor cells first developed into Ly6C^+^MHCII^-^ monocytes and subsequently into mature Ly6C⁻MHCII⁺ macrophages [18, 25]. Flow cytometry analysis revealed that CCN1 treatment significantly increased the proportion of Ly6C^−^MHCII^+^ mature macrophages to 48.1%, compared to only 1.78% in the untreated control group (Fig. 4B, C). The positive control group treated with L929 cell-conditioned medium exhibited enhanced differentiation, with Ly6C^-^MHCII^+^ cells reaching 75.8%, further confirming CCN1’s role in promoting macrophage maturation (Fig. 4B, C). CCK-8 assays further showed enhanced adherence of progenitor cells in the presence of CCN1 (Fig. 4D). These results indicate CCN1’s role in promoting macrophage differentiation and maturation. To further evaluate the effect of CCN1 on macrophage phenotype, flow cytometry analysis on BMDMs was performed (Supplementary Fig. 4A). The results revealed that CCN1 significantly increased Ly6C^-^ cells, but did not alter CD86^+^, CCR2^+^ or CD206^+^ populations (Fig. 4E, F), suggesting a potential modulation of pro-repair macrophage subsets.

Following the evaluation of CCN1’s impact on macrophage subset differentiation, we next investigated its effects on key macrophage functional properties, including migration and phagocytic capacity. To assess the effect of CCN1 on macrophage migration, BMDMs were stimulated with LPS and IFN-γ to mimic activation during AKI, and migration was evaluated under both stimulated and unstimulated conditions. Scratch wound assays demonstrated that CCN1 treatment significantly enhanced wound closure capacity in BMDMs (Supplementary Fig. 4B, C). Under basal conditions, CCN1 accelerated wound closure by 26.7 ± 1.7% at 24 h and 62.7 ± 14.5% at 48 h compared to untreated controls. This pro-migratory effect was maintained under LPS and IFN-γ stimulation, with CCN1-treated BMDMs showing 22.4 ± 2.8% and 46.5 ± 6.7% greater wound closure at 24 and 48 h, respectively, compared to stimulated controls. Consistent with these findings, Transwell migration assays demonstrated a significant increase in migrated BMDMs following CCN1 treatment after 24 h, regardless of LPS and IFN-γ stimulation (Supplementary Fig. 4D, E). These findings demonstrate that CCN1 promotes macrophage migration. To explore the effect of CCN1 on the phagocytosis of macrophages, FITC-labeled fluorescent latex beads were used to mimic cellular debris in the AKI environment. There was no significant difference in the proportion of FITC^+^ cells in CCN1-treated BMDMs compared with controls (Supplementary Fig. 4F, G), suggesting that CCN1 had no significant effect on phagocytosis of macrophages. Together, these findings indicate that CCN1 directly regulates macrophage differentiation and migration, highlighting its role in modulating macrophage function during AKI.

### CCN1-activated macrophages promote tubular epithelial proliferation

**Fig. 5:**
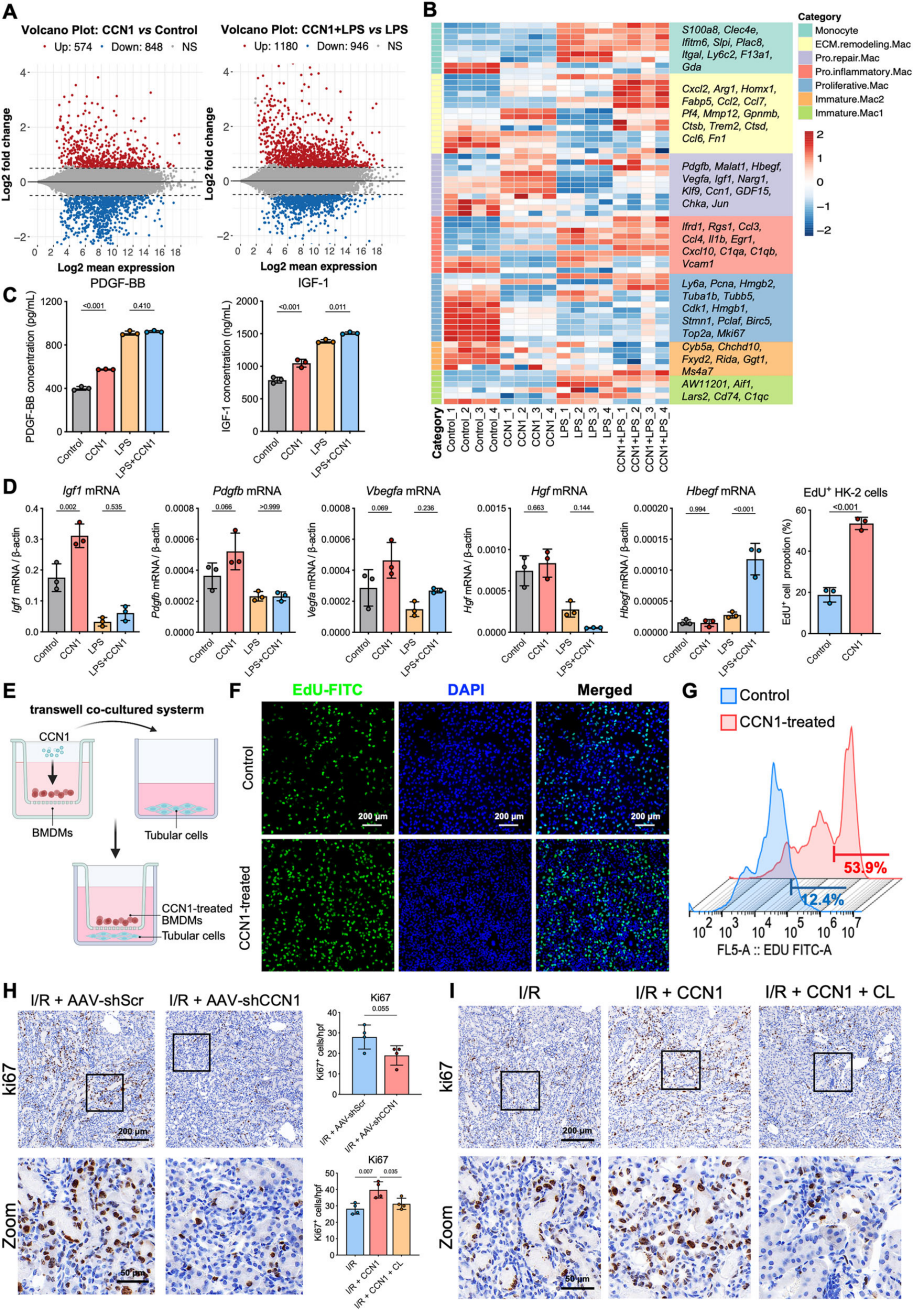
CCN1 promotes RTECs proliferation via macrophage regulation. **A** Volcano plot showing differentially expressed genes in BMDMs with or without CCN1 treatment under unstimulated or LPS and IFN-γ-stimulated conditions. **B** Heatmaps of marker genes of seven macrophage subpopulations for macrophage subpopulations. **C** Concentration of growth factors IGF-1 and PDGF-BB in BMDMs medium. **D** The mRNA expression of growth factors (*Igf1*, *Pdgfb*, *Vegfa*, *Hgf*, and *Hbegf*) in BMDMs. **E** A diagram of the transwell co-culture system with CCN1-treated BMDMs in the top chamber and HK-2 cells in the bottom chamber. **F** Representative image of EdU immunofluorescence staining and quantification of EdU^+^ proportion in HK-2 cells in the bottom chamber. **G** Flow cytometry analysis of EdU⁺ HK-2 cells in the co-culture system. *n* = 3 per group. **H** Representative images and quantitative analysis of Ki67 immunostaining in I/R mice kidney with AAV-shScr and AAV-shCCN1. **I** Representative images of Ki67 immunostaining in I/R mice kidney with CCN1 treatment and Clodronate Liposomes (CL). *n* = 4 mice per group. Data are presented as mean ± SD.

To further explore the potential mechanisms by which CCN1 regulates macrophage function, RNA-seq analysis was performed on BMDMs treated with or without CCN1 under both unstimulated and LPS and IFN-γ-stimulated conditions. Differential gene expression analysis revealed 1,422 differentially expressed genes (DEGs) in the absence of stimulation, including 574 upregulated genes and 848 downregulated genes. Under LPS and IFN-γ stimulation, there were 2126 DEGs, including 1180 upregulated genes and 946 downregulated genes (Fig. 5A). By integrating findings from previous single-cell spatial transcriptomic studies [14], we referenced marker genes associated with seven macrophage subpopulations identified during the AKI-to-CKD transition. CCN1 treatment upregulated genes related to “pro-repair macrophages” (*Pdgfb*, *Igf1*, *Hbegf*, *Vegfa*, *Malat1*, *Klf9*) and “ECM-remodeling macrophages” (*Arg1*, *Mmp12*, *Pf4*, *Fabp5*) (Fig. 5B). Conversely, genes associated with monocytes and immature macrophages (*Plac8*, *Ly6c2*, *F13a1*, *Gda*) were downregulated. However, the pro-inflammatory macrophage markers showed no significant change. To the effect of CCN1 protein on “pro-repair macrophages”, we detected growth factor levels in BMDMs. Interestingly, ELISA assays showed elevated secretion of growth factors from CCN1-treated BMDMs to the culture medium, with IGF-1 levels increasing from 700 ng/mL to 1000 ng/mL and PDGF-BB increasing from 400 pg/mL to 600 pg/mL (Fig. 5C). Meanwhile, compared with normal control BMDMs, CCN1 treatment specifically upregulated *Igf1* mRNA expression, while *Pdgfb* and *Vegfa* exhibited an upward trend but without statistical significance (Fig. 5D). However, no marked increase was detected in growth factors *Hgf* or *Hbegf* expression following CCN1 treatment (Fig. 5D).

In the pathophysiology of AKI, “pro-repair macrophages” actively contribute to kidney repair by promoting the proliferation of RTECs. To investigate the effect of CCN1 on macrophages-mediated proliferation of RTECs, we established a transwell co-culture system in which CCN1-treated BMDMs seeded in the top chamber and HK-2 cells in the bottom chamber (Fig. 5E). EdU immunofluorescence staining was used to assess the proliferative activity of HK-2 cells, and the data showed that CCN1-treated macrophages significantly increased the proportion of EdU⁺ proliferating HK-2 cells compared to normal macrophages (Fig. 5F). This pro-proliferative effect was quantitatively confirmed by flow cytometry, which demonstrated an increase from 12.4% to 52.9% in EdU^+^ HK-2 cells when co-cultured with CCN1-treated macrophages versus control macrophages (Fig. 5G). Interestingly, while direct stimulation of HK-2 cells with CCN1 alone could also slightly enhance their proliferation (Supplementary Fig. 5), the pro-proliferative effect was much more pronounced when mediated by CCN1-treated macrophages.

To validate these findings in vivo, we examined renal tubular epithelial proliferation in CCN1 knockdown mice, which displayed fewer Ki67^+^ tubular cells compared to control mice (Fig. 5H). To determine the dependence of CCN1-induced renal tubular epithelial proliferation on macrophages, we established a macrophage-depleted mouse model (Supplementary Fig. 6). In this model, CCN1 administration showed a markedly reduced ability to promote tubular epithelial cell proliferation compared with control mice, indicating that macrophages are essential mediators of CCN1’s pro-proliferative effects (Fig. 5I). These results reveal that CCN1 may promote the proliferation of RTECs by regulating macrophage phenotype.

### Deconvolution analysis identifies CCN1-induced ARG1^+^ pro-repair macrophages

**Fig. 6:**
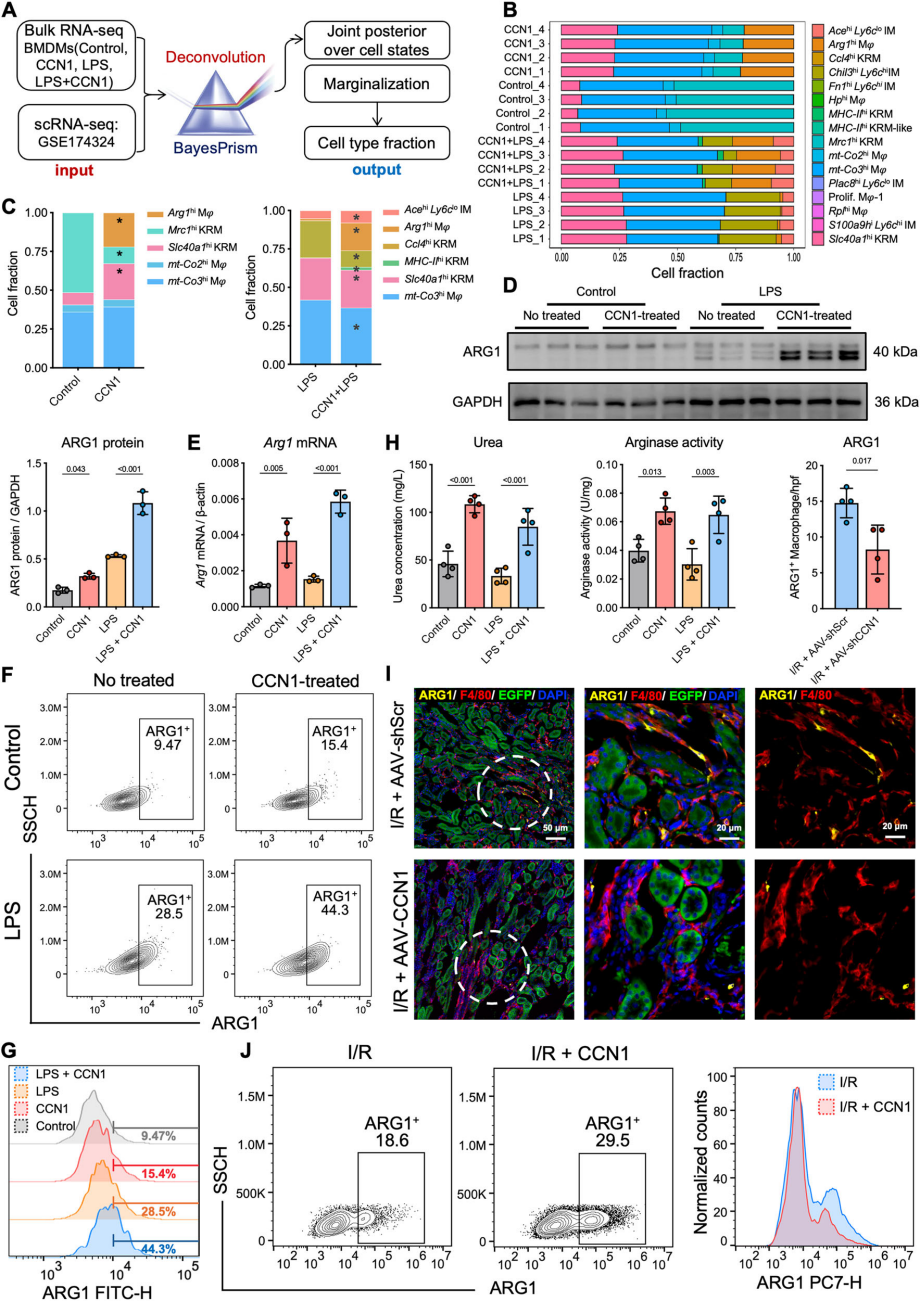
Deconvolution analysis identifies CCN1-induced ARG1^+^ macrophages. **A** BayesPrism workflow of scRNA-seq (GSE174324) and bulk RNA-seq transcriptomic data integration and deconvolution based on BayesPrism to infer joint. **B** Cell-type fraction of deconvolution analysis across all samples. **C** Comparison of cell-type fractions following CCN1 treatment under normal and LPS-stimulated conditions. *n* = 4 per group. ^*^P < 0.05. **D**, **E** The protein and mRNA expression of ARG1 in BMDMs. **F**, **G** Contour plot and histogram of flow cytometry of the proportion of ARG1^+^ cells in BMDMs. **H** The concentration of urea and Arginase activity in BMDMs. *n* = 4 per group. Data are presented as mean ± SD. **I** Representative fluorescent micrographs and quantification analysis of ARG1 (yellow), F4/80 (red) and EGFP-tag staining in I/R mice kidney with AAV-shScr and AAV-shCCN1 injection. **J** Flow cytometry of I/R mice kidney with CCN1 treatment. *n* = 4 mice per group. Data are presented as mean ± SD.

To identify underlying cell subtypes, we used BayesPrism [26] to deconvolute bulk RNA-seq to study (Supplementary Table 1). The Bayesian statistical model jointly infers cell-type composition and cell-type-specific gene expression profiles within the bulk RNA-seq count matrix (Fig. 6A). Using this technique, we demonstrated that under unstimulated conditions, CCN1-treated BMDMs primarily comprised five subpopulations: *Arg1*^hi^, *Mrc1*^hi^, *Slc40a1*^hi^, *mt-Co2*^hi^, and *mt-Co3*^hi^. Under LPS and IFN-γ stimulation, six subpopulations were identified: *Ace*^hi^ *Ly6c*^lo^ IM, *Arg1*^hi^, *Ccl4*^hi^ KRM, *MHC-II*^hi^ KRM, *Slc40a1*^hi^ KRM, and *mt-Co3*^hi^ Macrophage (Fig. 6A). Notably, *Arg1*^hi^ macrophages were increased in both conditions following CCN1 treatment (Fig. 6C). Together, these findings reveal that CCN1 promotes *Arg1*^hi^ macrophages.

To validate the RNA-seq results, we examined ARG1 expression in BMDMs after CCN1 protein treatment. Western blot and qRT-PCR analyses confirmed a significant increase in ARG1 protein and *Arg1* mRNA expression in CCN1-treated BMDMs (Fig. 6D, E). Flow cytometry analysis showed that CCN1 significantly increased the proportion of ARG1^+^ cells from 9.47% to 15.4%, and CCN1 increased this proportion from 28.5% to 44.3% under LPS and IFN-γ stimulation conditions (Fig. 6F, G). Consistently, CCN1-stimulated macrophages exhibited significantly elevated ARG1 activity as well as urea levels, indicating that CCN1 promotes ARG1-mediated metabolic activity (Fig. 6H). In vivo, ARG1 was upregulated in F4/80⁺ macrophages after I/R-AKI, and these ARG1⁺ macrophages were often adjacent to CCN1-high RTECs, consistent with CCN1’s role in promoting ARG1^+^ macrophage in vivo. (Supplementary Fig. 7). In the tubule-specific CCN1 knockdown mouse model, ARG1 and F4/80 co-immunofluorescence staining showed that ARG1^+^ macrophages were significantly reduced (Fig. 6I). These results indicated that CCN1 knockdown reduced ARG1^+^ macrophages. Conversely, administration of CCN1 protein significantly increased ARG1^+^ macrophages, as confirmed by flow cytometry (Fig. 6J). Collectively, these findings establish that CCN1 promotes the differentiation and accumulation of ARG1^+^ pro-repair macrophages both in vitro and in vivo.

### ARG1 mediates CCN1-driven macrophage promotion of tubular epithelial proliferation

**Fig. 7:**
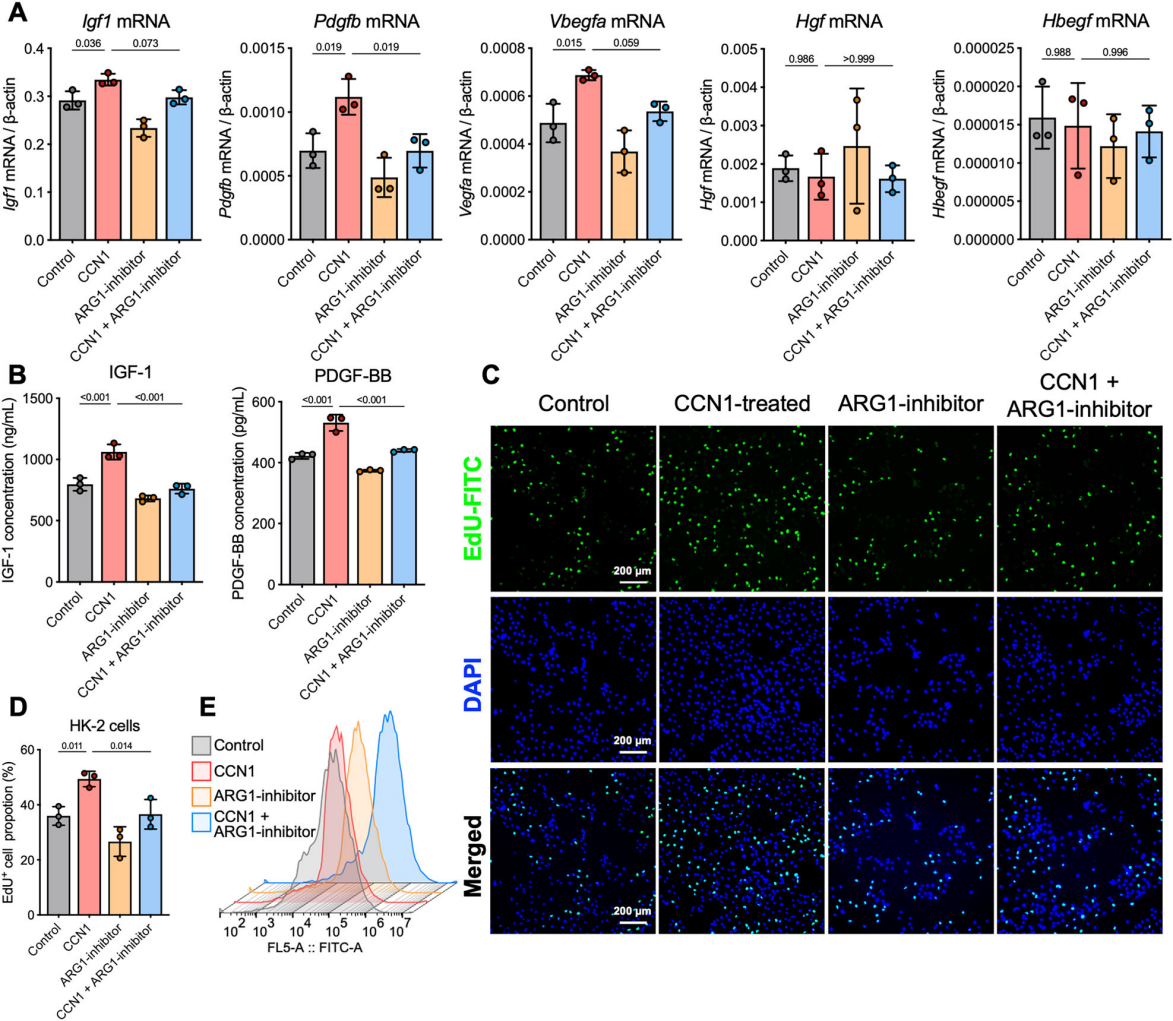
CCN1 promotes macrophage-mediated proliferation of RTECs through ARG1. **A** mRNA expression of growth factor gene (*Igf1*, *Pdgfb*, *Vegfa, Hgf, and Hbegf*) in BMDMs. **B** Concentration of IGF-1 and PDGF-BB in BMDMs medium. **C**, **D** Representative image of EdU immunofluorescence staining and quantification of EdU^+^ proportion in HK-2 cells in the bottom chamber. **E** Flow cytometric analysis of EdU^+^ HK-2 cells in the co-culture system. *n* = 3 per group. Data are presented as mean ± SD.

Building on established roles of *Arg1*^hi^ macrophages in promoting RTECs proliferation in AKI, we investigated whether CCN1 facilitates this process via ARG1 using Numidargistat, a pharmacological inhibitor of ARG1. The results showed that mRNA levels of growth factors *Igf1*, *Pdgfb*, and *Vegfa* were elevated in CCN1-treated BMDMs, but this upregulation was significantly inhibited by the addition of Numidargistat (Fig. 7A). In addition, Numidargistat also suppressed the concentration of growth factors IGF-1 and PDGF-BB released from CCN1-treated BMDMs (Fig. 7B). In a transwell co-culture assay, CCN1-induced increase in EdU^+^ proliferating cells in HK-2 cells co-cultured with BMDMs was inhibited when Numidargistat was added (Fig. 7C, D). Flow cytometry analysis yielded consistent results, indicating that CCN1 and ARG1 inhibitor co-treated BMDMs significantly decreased the proportion of EdU^+^ HK-2 cells compared with CCN1 alone treatment (Fig. 7E). These findings reveal that CCN1 enhances RTECs proliferation through the regulation of macrophages in an ARG1-dependent manner.

### CCN1 activates macrophage STAT6/ARG1 pathway via α_v_β_5_ to promote tubular epithelial proliferation

**Fig. 8:**
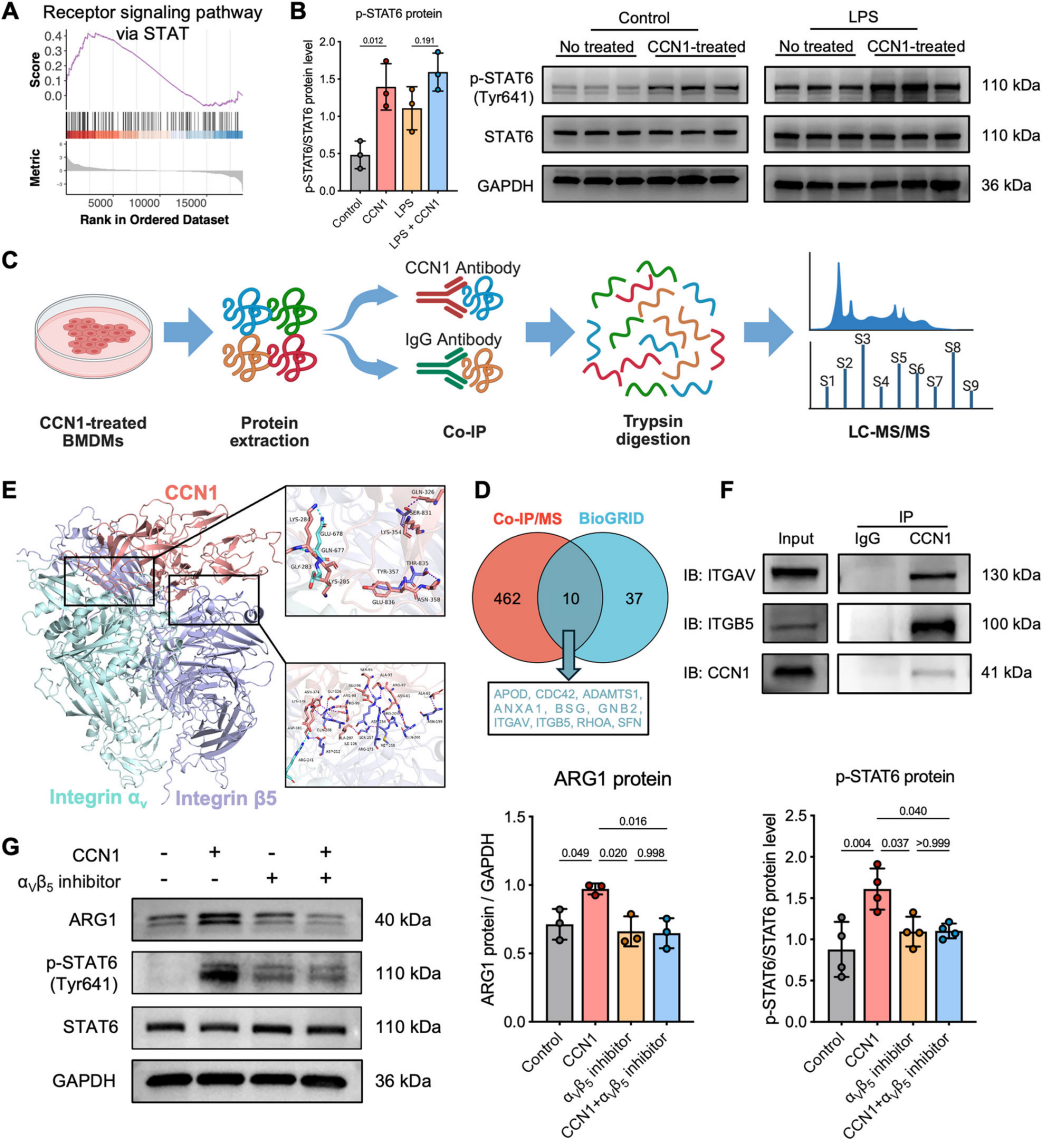
CCN1 activates the STAT6/ARG1 pathway in macrophages via α_v_β_5_ integrin. **A** GSEA enrichment of STAT-related pathways in CCN1-treated BMDMs. **B** Protein levels of p-STAT6, STAT6 and ARG1 in BMDMs. **C** Co-IP and MS workflow for CCN1-interacting proteins. **D** Overlap of CCN1-interacting proteins identified by Co-IP/MS and BioGRID database. **E** Molecular docking of CCN1 with ITGAV and ITGB5. **F** Co-IP validation of CCN1 binding to ITGAV and ITGB5 in BMDMs. **G** Protein levels of p-STAT6, STAT6 and ARG1 after α_v_β_5_ integrin inhibitor treatment. *n* = 3 per group. Data are presented as mean ± SD.

To elucidate how CCN1 enhances ARG1 expression, we performed GSEA enrichment analysis, which revealed that STAT-related pathways were activated in macrophages treated with CCN1 protein (Fig. 8A). Since STAT6 is a key upstream transcription factor of ARG1, we hypothesized that CCN1 activates the STAT6/ARG1 pathway. Consistently, western blot analysis showed that CCN1 stimulation markedly increased STAT6 phosphorylation in BMDMs (Fig. 8B), indicating activation of the STAT6/ARG1 pathway. To further investigate how secreted CCN1 mediates tubular-macrophage communication, we performed co-immunoprecipitation (Co-IP) with mass spectrometry (MS) to identify CCN1-binding proteins in macrophages (Fig. 8C). Integration of MS results with the BioGRID database (https://thebiogrid.org) revealed 10 potential interactors, including integrins ITGAV and ITGB5, which are broadly expressed on the macrophage surface (Fig. 8D). Molecular docking suggested that CCN1 can stably bind to ITGAV and ITGB5 (Fig. 8E). This interaction was further confirmed by Co-IP in BMDMs, demonstrating their binding in cells (Fig. 8F). Moreover, treatment with an α_v_β_5_ integrin inhibitor significantly suppressed STAT6 phosphorylation and ARG1 expression, confirming the functional role of α_v_β_5_ integrin in CCN1-mediated STAT6/ARG1 activation (Fig. 8G). Collectively, these results indicate that CCN1 regulates macrophage-mediated repair following AKI via the α_v_β_5_-STAT6/ARG1 signaling pathway.

## Disscussion

This study demonstrates that RTECs upregulate and secrete CCN1 in response to I/R injury. CCN1 regulates macrophage function to promote RTEC proliferation, a process mediated through the α_v_β_5_-STAT6-ARG1 signaling axis. Our findings reveal a novel tubular-immune crosstalk mechanism critical for kidney repair following ischemic injury and identify CCN1 as a potential therapeutic target for AKI.

CCN1 is a secreted matricellular protein that is indispensable for cardiovascular development during embryogenesis and is involved in inflammation, wound healing, and tissue repair in adulthood. Recent studies have highlighted the role of CCN1 in promoting tissue regeneration, such as enhancing cutaneous wound healing and liver regeneration [16, 27, 28]. Our previous work showed that CCN1 is upregulated in the early stages of AKI and serves as a potential early biomarker [22]. Building on these observations, the current study demonstrates that CCN1-high RTECs are spatially adjacent to F4/80^+^ macrophages in injured kidneys, suggesting functional paracrine interactions. To establish CCN1’s causal role, we employed complementary gain-of-function and loss-of-function approaches. Administration of recombinant CCN1 protein ameliorated kidney injury, reduced tubular damage, and increased renal macrophage infiltration, particularly the Ly6C^−^ subset. Conversely, RTEC-specific CCN1 knockdown via adeno-associated virus exacerbated I/R-AKI, decreased renal macrophages, and reduced tubular epithelial proliferation. These bidirectional results establish that tubular-derived CCN1 is not merely a biomarker but an active regulator of kidney repair.

A key finding of this study is the identification of macrophages as the primary cellular target mediating CCN1’s protective effects. RNA sequencing of kidney tissues from CCN1-treated mice revealed enrichment of immune-related pathways, particularly macrophage activation and immune response mediator production, indicating that CCN1’s in vivo effects are predominantly immune-mediated rather than direct actions on parenchymal cells. This interpretation is further supported by several lines of evidence. First, while CCN1 modestly increased tubular epithelial cell proliferation in vitro in the absence of macrophages, this direct effect was substantially weaker than the robust proliferation observed in co-culture with CCN1-treated macrophages. Second, and most critically, macrophage depletion using clodronate liposomes abolished CCN1’s ability to promote tubular epithelial proliferation in vivo, demonstrating the essential role of macrophages in mediating CCN1’s protective effects. Together, these findings indicate that CCN1 functions primarily through macrophage activation to drive kidney repair, with direct effects on tubular cells playing a minor role.

To elucidate the specific macrophage populations regulated by CCN1, we performed RNA-seq on CCN1-treated BMDMs and applied BayesPrism deconvolution using the publicly available scRNA-seq dataset GSE174324 as the reference. This dataset, derived from mononuclear phagocytic cells isolated from mouse kidney, blood, and spleen at baseline, day 1 and day 3 following unilateral renal I/R injury, defines 22 distinct monocyte/macrophage subpopulations, including kidney-resident macrophages and infiltrating macrophages [29]. Deconvolution analysis revealed that CCN1 treatment increased *Arg1*^hi^ macrophages under both basal and stimulated conditions. Liu et al. previously conducted subcluster analysis of myeloid cells from kidneys harvested at various time points (day 1–28) after I/R injury and identified 11 distinct macrophage subpopulations. Among them, pro-repair macrophages emerged early (day 1–3) and progressively declined during the chronic phase [14]. By integrating the signature genes of these macrophage clusters with our RNA-seq data from CCN1-treated BMDMs, we found that CCN1 modulated gene expression in at least seven macrophage clusters. Notably, genes associated with tissue repair, such as *Pdgfb*, *Igf1*, and *Hbegf*, were significantly upregulated. This temporal correlation between high CCN1 expression and the emergence of pro-repair macrophages in early I/R-AKI suggests a potential role for CCN1 in orchestrating macrophage-mediated kidney repair.

Functionally, we demonstrate that CCN1-induced *Arg1*^hi^ macrophages promote tubular epithelial cell proliferation through paracrine signaling. ARG1, a key arginase, catalyzes the conversion of L-arginine to L-ornithine and urea, a metabolic process associated with tissue repair and immunomodulation [30, 31]. We confirmed functional ARG1 activity by measuring arginase enzymatic activity and urea production in CCN1-treated macrophages, both of which were significantly elevated. Pharmacological inhibition of ARG1 or genetic knockdown abolished CCN1-treated macrophages’ ability to promote tubular epithelial proliferation in vitro, and ARG1 inhibition attenuated CCN1’s protective effects in vivo. These findings establish ARG1 as a critical downstream effector of CCN1-mediated kidney repair. Furthermore, CCN1-treated macrophages displayed enhanced expression and secretion of growth factors, including PDGF-BB and IGF-1, which are known to accelerate epithelial repair following AKI [32–34]. In our in vitro experiments, CCN1-treated macrophages displayed enhanced expression and secretion of growth factors, including PDGF-BB and IGF-1. Furthermore, Transwell co-culture assays revealed that CCN1-treated macrophages significantly promoted the proliferation of underlying RTECs. It is widely accepted that epithelial repair after AKI involves dedifferentiation, migration, and proliferation of surviving RTECs, processes facilitated by growth factors such as HGF, IGF-1, and EGF family members [35]. Our findings suggest that CCN1-activated macrophages contribute to this regenerative niche by secreting multiple growth factors that collectively drive tubular repair.

Mechanistically, we identified the α_v_β_5_ integrin-STAT6-ARG1 signaling axis as the pathway through which CCN1 regulates macrophage function. GSEA of CCN1-treated macrophages revealed activation of STAT-related pathways, and western blot analysis confirmed increased STAT6 phosphorylation and ARG1 expression. Co-immunoprecipitation coupled with mass spectrometry identified integrin α_v_β_5_ as a direct CCN1-binding partner on macrophages, an interaction validated by molecular docking and Co-IP experiments. Pharmacological inhibition of α_v_β_5_ integrin suppressed CCN1-induced STAT6 phosphorylation and ARG1 expression, establishing the functional requirement of this receptor for CCN1’s effects. STAT6 is a well-established transcription factor regulating ARG1 expression in alternatively activated macrophages [36, 37], and our findings extend this pathway by demonstrating that extracellular CCN1 initiates this signaling cascade through integrin engagement. This discovery provides molecular insights into how RTECs communicate with infiltrating macrophages to coordinate tissue repair.

The clinical relevance of our findings is supported by analysis of single-cell RNA-seq data from the Kidney Precision Medicine Project [38], which revealed that CCN1 expression is significantly elevated in kidney tissues from AKI patients compared with healthy controls, with predominant expression in RTECs. This observation aligns with our murine data and suggests that the CCN1-macrophage axis may operate in human AKI. Moreover, the spatial proximity of CCN1-high tubules to Arg1^+^ macrophages in both mouse and human injured kidneys supports the translational potential of targeting this pathway therapeutically.

Several limitations warrant consideration. First, while AAV-mediated RTEC-specific CCN1 knockdown achieved targeted suppression, a constitutive conditional knockout model would provide more definitive genetic evidence. Second, while BayesPrism deconvolution successfully identified macrophage subpopulation changes, direct single-cell RNA sequencing of kidney immune cells from CCN1-treated or knockdown mice would provide higher resolution. Finally, detailed characterization of T cell subsets and their interactions with CCN1-activated macrophages represents an important area for future investigation.

In conclusion, this study reveals a novel tubular-immune communication pathway in which renal tubular epithelial cell-derived CCN1 engages α_v_β_5_ integrin on infiltrating macrophages to activate the STAT6-ARG1 axis, promoting macrophage-mediated tissue repair and tubular epithelial proliferation following I/R-AKI. These findings not only advance our understanding of the cellular and molecular mechanisms governing kidney repair but also identify CCN1, α_v_β_5_ integrin, and the STAT6-ARG1 pathway as potential therapeutic targets for ischemic AKI. Future studies investigating CCN1-based therapeutic strategies, including recombinant protein administration or small molecule agonists targeting the CCN1-α_v_β_5_-STAT6-ARG1 axis, may hold promise for accelerating kidney recovery and improving clinical outcomes in AKI patients.

## Materials and methods

### Mice

Male C57BL/6 mice (6-8 weeks old) were purchased from Pengyue Laboratory Animal Center (Jinan, China). The mice were housed in a specific pathogen-free environment at the experimental animal platform of the Biomedical Center of Qingdao University at the optimal temperature with a 12 h light/12 h dark cycle. Sample sizes were based on standard protocols in the field. Mice were randomly assigned to groups in experiments, but no blinding was performed during animal experiments or the isolation of primary cells. All animal experiments were performed in strict accordance with the guidelines of the National Institutes of Health Guide for the Care and Use of Laboratory Animals and were approved by the Animal Ethics Committee of Qingdao University (20240401C571620240501105).

### I/R-AKI model

A unilateral I/R model was established by right nephrectomy followed by contralateral renal pedicle clamping for 18 minutes with 4 mice per group. Mice were anesthetized with isoflurane. Seven days after right nephrectomy, the left renal pedicle was clamped using a non-traumatic microaneurysm clip for 18 minutes to induce ischemia, followed by reperfusion. During the ischemia period, the body temperature of mice was maintained at 38°C. Mice were euthanized on day 1, day 2, or day 3 after unilateral I/R. At the end of the experiment, mice were sacrificed by inhalation of carbon dioxide under isoflurane anesthesia. Blood and kidney tissues were collected for subsequent analyses. Blood samples were used to assess renal function and kidney injury biomarkers, while kidney tissues were analyzed for histopathological changes and related molecular markers.

## Renal function assessment

Blood samples were obtained from the orbital sinus to assess renal function. Serum creatinine and blood urea nitrogen were detected by renal function detection kits (E-BC-K188-M; E-BC-K183-M, Elabscience, Wuhan, China) following the product manual.

### Histological analysis

Kidney tissues were fixed in 4% paraformaldehyde, embedded in paraffin, and cut into 4-μm sections. Sections were stained with H&E reagents. Histological scoring was performed under a light microscope in a blinded manner based on tubular injury severity (loss of brush border, tubular dilation, cast formation, and necrosis) [39]. At least 10 randomly selected fields per sample were assessed, and injury scores were assigned as follows: 0 (normal), 1 (<10%), 2 (10–25%), 3 (26–50%), 4 (51–75%), and 5 (>75%).

### Immunohistochemistry

Deparaffinized kidney tissue sections underwent antigen retrieval using citrate buffer (pH 6.0), and endogenous peroxidase activity was blocked with 3% hydrogen peroxide. After blocking with 5% BSA, sections were incubated overnight with KIM1 (1:100, #14971), F4/80 (1:500, #70076), CCN1 (1:50, #39382) and Ki67 (1:200, #12202) primary antibodies (all from Cell Signaling Technology, Danvers, MA, USA) at 4 °C. The next day, sections were incubated with HRP-conjugated secondary antibodies and developed using DAB substrate [40]. Nuclei were counterstained with hematoxylin. Images were captured with a light microscope (Nikon, Tokyo, Japan).

### Western blot analysis

Total proteins were extracted from kidney tissues or cells using RIPA lysis buffer containing protease and phosphatase inhibitors. Protein concentrations were determined using the BCA Protein Assay Kit (Elabscience). Equal amounts of protein (30–50 μg) were separated by SDS-PAGE and transferred onto PVDF membranes (Millipore, Burlington, MA, USA). Membranes were blocked with 5% non-fat milk or 5% BSA for 1 h at room temperature and incubated overnight at 4°C with CCN1 (#39382), ARG1 (#93668), Phospho-STAT6 (Tyr641) (#9361), STAT6 (#5397) and GAPDH (#2118) primary antibodies (all 1:1000, Cell Signaling Technology). After washing, membranes were incubated with HRP-conjugated secondary antibodies for 1 h at room temperature. Protein bands were visualized using enhanced chemiluminescence (ECL) reagents (Elabscience) and quantified with ImageJ software [41].

### qRT-PCR

Total RNA from kidney tissue, HK-2 cells, or BMDMs was extracted using TRIzol reagent (Invitrogen, Thermo Fisher Scientific, Waltham, MA, USA). One microgram of RNA was reverse transcribed into cDNA using a commercial kit. qRT-PCR was performed using SYBR Green Master Mix on a Bio-Rad CFX96 Real-Time PCR system (Bio-Rad, Hercules, CA, USA) [42]. Gene expression levels were normalized to β-actin and calculated using the 2^-ΔCT^ method. Primer sequences are listed in Supplementary Table 2.

### ELISA

Mouse serum samples and cell culture supernatants were centrifuged for 10 minutes at 3000 rpm. The levels of CCN1 in serum and HK-2 cell culture supernatants, as well as PDGF-BB, IGF-1 of BMDMs supernatants were measured using ELISA kits (Elabscience) according to the manufacturers’ instructions [43].

### Immunofluorescence staining

Paraffin-embedded kidney sections were deparaffinized, rehydrated, and subjected to antigen retrieval in citrate buffer (pH 6.0). After blocking with 10% normal goat serum, sections were incubated overnight at 4 °C with CCN1 (1:50, #39328), AQP1 (1:50, #69343), F4/80 (1:200, #30325) or ARG1 (1:50, #93668) primary antibodies (all from Cell Signaling Technology), followed by fluorophore-conjugated secondary antibodies [44]. Nuclei were counterstained with DAPI. Images were acquired using a confocal microscope (Nikon).

### Mice CCN1 recombinant protein injection

An I/R mouse model was established by right nephrectomy and left kidney clamping for 18 min. CCN1 recombinant protein 0.2 mg/kg was injected through the tail vein immediately after the operation, and samples were collected 3 days after the operation, with four mice per group.

### RNA-seq

Total RNA from Kidney tissues and BMDMs was extracted using TRIzol reagent (Invitrogen) and treated with DNase I (Takara, Shiga, Japan). RNA quality was assessed using the Agilent 2100 Bioanalyzer, and concentration was measured using a NanoDrop ND-2000 spectrophotometer. Only high-quality RNA samples (OD260/280 = 1.8–2.2, OD260/230 ≥ 2.0, RIN ≥ 6.5, 28S:18S ≥ 1.0, >1 μg) were used for library preparation. RNA purification, reverse transcription, library construction, and sequencing were performed by BGI (Shenzhen, China) using the Illumina HiSeq XTen platform.

Data were analyzed using Python and R. Transcript abundance was quantified as fragments per kilobase of exon per million mapped reads (FPKM) using RSEM. DEGs were identified using DESeq2 with thresholds of the absolute value of log_2_ fold change ≥1 and adjusted *P* value < 0.05. Gene set enrichment analysis (GSEA) was performed using the msigdb.v2022.1.Mm.symbols.gmt gene sets, and enrichment scores were calculated to identify significantly enriched pathways. Volcano plots and heatmaps of DEGs were generated using R to visualize expression patterns and sample clustering. BayesPrism was performed following the original paper’s pipeline [26], using GSE174324 [29] as the reference single-cell RNA-seq dataset, which contains 22 monocyte/macrophage subpopulations from mouse kidney, blood, and spleen at baseline and post-ischemia-reperfusion injury (days 1 and 3). Key subpopulations include C1 *MHC-II*^hi^ KRM, C3 *Mrc1*^hi^ KRM, C5 *Arg1*^hi^ Mф, and C6 *S100a9*^hi^ *Ly6C*^hi^ IM Bulk RNA-seq data from BMDMs were normalized to TPM and deconvoluted using the GSE174324 expression matrix and cell-type annotations with default BayesPrism parameters.

### Flow cytometry

#### Kidney cell preparation

The mouse kidney was dissected, and the capsule was removed. The tissue was minced on a plate containing collagenase solution (130-110-203, Miltenyi Biotec, Bergisch Gladbach, Germany) and digested at 37°C with agitation for 30 minutes. The homogenate was passed through a 70 µm cell strainer to obtain a single-cell suspension. The suspension was centrifuged at 1200 rpm for 5 minutes, and the resulting pellet was washed with PBS. Cells were then resuspended in 5 mL of red blood cell lysis buffer, incubated for 5 minutes, and centrifuged again at 1200 rpm for 5 minutes. Cells were washed with PBS and centrifuged for subsequent staining.

#### BMDMs preparation

Cells were collected using PBS containing 1 mM EDTA and placed on ice for 5 minutes. Cells were washed with PBS and centrifuged for staining.

#### Cell staining

Cells were resuspended in 200 μL PBS with 1% BSA and incubated with 2 μL anti-CD16/32 antibody at room temperature for 10 minutes. For kidney cells, cells were stained with antibodies against CD45, CD11b, F4/80, CD86, CCR2, Ly6C, CD3, CD8, CD4 and Fixable Viability Stain 700 (FVS700) for 15 minutes in the dark. For BMDMs, cells were stained with antibodies against CD11b, F4/80, CD86, CCR2, and Ly6C for 15 minutes in the dark. Cells were fixed with 250 μL fixation buffer (#420801, Biolegend, San Diego, CA, USA), permeabilized with intracellular permeabilization buffer (#421002, Biolegend), and stained with CD206 and ARG1 antibody for 15 minutes in the dark. For macrophage maturation analysis, BMDMs were 1 μL of MHC-II and Ly6C antibodies were added and incubated in the dark for 15 minutes. Then cells were washed and analyzed using a CytoFlex S flow cytometer. FlowJo software (v10.8.1) was used for quantitative analysis and figure generation. Flow cytomety-related antibodies are all provided in Supplementary Table 3.

### Adeno-associated virus mouse model

To investigate the role of CCN1 in kidney injury, tubular-specific CCN1 knockdown mice were generated using AAV with 4 mice per group. shRNA targeting *Ccn1* (#1: AGCTTCCAGCCCAACTGTAAA, #2: TGGAGTTAACGAGAAACAATG) (AAV-shCCN1) or a scramble control (TTCTCCGAACGTGTCACGT) (AAV-shScr) was cloned into GV909 (pAAV-Kspcadherin-EGFP-MCS) via NheI and XhoI restriction sites and verified by DNA sequencing (Genechem Co., Ltd., Shanghai, China). Four weeks prior to I/R model construction, mice were injected with 50 μL of AAV-shCCN1 or AAV-shScr (5 × 10^10^ vg/mL) into the left kidney via the renal pelvis.

### Cell isolation and culture

HK-2 and BMDMs were used in this study. HK-2 cells were purchased from Procell (Wuhan, China), authenticated by short tandem repeat (STR) profiling, and cultured in Dulbecco’s Modified Eagle Medium/Nutrient Mixture F-12 (DMEM/F12; Hyclone, GE Healthcare Life Science, Logan, UT, USA) supplemented with 10% fetal bovine serum (ScienCell, San Diego, CA, USA) and 1% penicillin-streptomycin (Gibco, Grand Island, NY, USA). BMDMs were generated by isolating bone marrow cells from the femurs and tibias of C57BL/6 mice and seeding them in non-tissue culture-treated dishes or plates. The cells were cultured for 7 days in DMEM/F12 medium supplemented with 10% L929 cell-conditioned medium, 10% fetal bovine serum, and antibiotics to promote differentiation into mature macrophages. All cells were maintained at 37 °C in a humidified incubator containing 5% CO_2_.

### In vitro renal tubular injury models

For the H/R model, HK-2 cells were cultured in glucose- and serum-free medium and placed in a hypoxia chamber (5% CO_2_, 1% O_2_, 94% N_2_) for 24 h, followed by 3 h of reoxygenation [45]. For the H_2_O_2_ model, HK-2 cells were treated with 500 nM H_2_O_2_ in fresh medium for 24 h. Control cells were cultured in normoxic conditions with fresh medium for the same duration.

### CCN1 recombinant protein stimulation

To evaluate macrophage maturation, bone marrow-derived cells were seeded in 6-well non-TC-treated plates. The control group received standard culture medium (DMEM/F12 with 10% FBS and antibiotics), while the experimental group was treated with 2 μg/mL CCN1 recombinant protein (MedChemExpress, Monmouth Junction, NJ, USA). The positive control group was supplemented with 10% L929 cell-conditioned medium. After 7 days of incubation, macrophage maturation was assessed. For other functional assays, BMDMs were seeded in TC-treated six-well plates and treated for 24 h with 2 μg/mL CCN1 recombinant protein. Cells were then harvested for further analyses.

### Scratch assay

A scratch was made in a confluent monolayer of BMDMs using a 1-mL pipette tip. Floating cells were removed by washing, and the scratch area was imaged every 24 h under a microscope. The scratch width was measured using ImageJ software (v1.53a).

### Transwell migration assay

Transwell assays were performed using 8-μm pore size inserts (Corning® Costar® Transwell®, Corning Inc., Corning, NY, USA). A total of 2 × 10^5^ BMDMs were suspended in 200 μL serum-free DMEM and seeded in the upper chamber, while 700 μL serum-free DMEM was added to the bottom chamber. Cells were allowed to adhere for 12 h at 37 °C. The bottom of the insert was swabbed to remove migrated cells, and 10 ng/mL CCL2 was added to the bottom chamber with 2 μg/mL CCN1 protein in the upper chamber. After a further 12-h incubation, migrated cells were fixed with 4% paraformaldehyde, washed, and stained with 0.1% crystal violet (Invitrogen) for 15 minutes. Images were captured using a Nikon Eclipse Ti2 microscope at ×10 magnification.

### Phagocytosis assay

According to the phagocytosis kit instructions, latex bead-rabbit IgG-FITC complexes were diluted 1:100 in pre-warmed culture medium and incubated with cells at 37°C for 3 h. Cells were washed three times with PBS and observed under a fluorescence microscope. Cells were then suspended in FACS buffer and analyzed by flow cytometry.

### Co-culture assay

HK-2 cells were seeded in the bottom chamber of a 12-well Transwell system. BMDMs were seeded in the top chamber and treated with 2 μg/mL CCN1 protein, 50 μM ARG1 inhibitor (Numidargistat, MedChemExpress), or a combination of both. Control cells received standard medium. After 24 h, the top chamber medium was replaced with fresh medium, and co-culture was continued for another 24 h before downstream analyses.

### EdU proliferation assay

After treatment, cells were washed with PBS and incubated with 10 μM EdU labeling reagent. Cells were then fixed, permeabilized, and stained using Click-iT reaction cocktail and DAPI according to the manufacturer’s protocol. EdU-positive cells were visualized under a fluorescence microscope and quantified using ImageJ.

### LDH and CCK-8 assays

To assess cytotoxicity, cell culture supernatants were collected. The positive control group was treated with 2% Triton X-100 for 30 minutes. Fifty microliters of each supernatant sample were mixed with 50 μL LDH assay reagent (#4744934001, Roche, Mannheim, Germany) in 96-well plates, and absorbance was measured at 450 nm every 5 minutes. Cell viability was evaluated using the CCK-8 assay. After washing, cells were incubated with fresh medium containing CCK-8 reagent (MedChemExpress) for 1 h, and absorbance was measured at 450 nm.

### Arginase activity and urea measurement

Arginase activity and urea production in cells or tissue lysates were measured using commercial microplate assay kits (Urea Microplate Assay Kit, Cat. No. abs580144, and Arginase Microplate Assay Kit, Cat. No. abs580116, Absin, Shanghai, China) according to the manufacturers’ instructions. Briefly, samples were incubated with the provided reagents, and the resulting colorimetric reactions were measured at 525 nm for arginase activity and 620 nm for urea concentration using a microplate reader. Urea levels were quantified based on a standard curve and expressed as mg/L, while arginase activity was calculated according to the generated urea and expressed as U/mg protein.

### Co-IP and MS analysis

Cells or tissue samples were lysed in IP lysis buffer supplemented with protease and phosphatase inhibitors. Lysates were incubated on ice for 30 minutes and centrifuged at 12,000 × *g* for 10 minutes at 4 °C to remove debris. Protein concentration was determined using a BCA assay. Equal amounts of protein were incubated overnight at 4 °C with a specific antibody against CCN1 or control IgG, followed by incubation with magnetic beads for overnight at 4 °C. Beads were washed three times with lysis buffer, and immunocomplexes were eluted by boiling in SDS loading buffer for SDS-PAGE and Western blot analysis. For mass spectrometry analysis, immunoprecipitated proteins were separated by SDS-PAGE, coomassie brilliant blue stained, and gel bands were excised, digested with trypsin, and analyzed by LC-MS/MS. The relative abundance of proteins co-precipitated with CCN1 was compared to that with the IgG control to identify specific interacting proteins. Reciprocal Co-IP confirmed interactions between CCN1 and ITGAV, ITGB5, and a Western blot was used to detect the corresponding binding partners.

### AlphaFold3-based prediction of CCN1-α_v_β_5_ interaction

The structural interaction between CCN1 and integrin α_v_β_5_ was predicted using AlphaFold3 (https://alphafoldserver.com). Amino acid sequences of CCN1, ITGAV, and ITGB5 were obtained from the UniProt database. Protein-protein docking models were generated using the multimer mode of AlphaFold3, and the top-ranking predicted complexes were analyzed for interface residues and binding regions. Structural visualization and analysis were performed using PyMOL (Schrödinger, San Diego, CA, USA).

### Statistical analysis

All data are presented as the mean ± SD from at least three independent experiments. All measurements were taken from distinct samples, and no data were excluded. Statistical analyses were performed using GraphPad Prism (v9.0). A normality test and an F test were performed on all data prior to parametric analysis. For comparisons between two groups, the Student’s *t* test was used. For multiple group comparisons, one-way or two-way ANOVA followed by Bonferroni’s post hoc test was applied. A *P*-value < 0.05 was considered statistically significant.

## Supplementary information

Supplementary materials

Original Western blots

## Data Availability

The datasets generated and/or analysed during the current study are available from the corresponding author on reasonable request.
