# Supplementary material for: Tubule-derived CCN1 drives renal repair via αvβ5-STAT6-ARG1-dependent reprogramming of macrophages
Source: Cell Death Dis. 2025 Dec 21;17(1):115. doi: 10.1038/s41419-025-08340-2 (PMC12847786; doi:10.1038/s41419-025-08340-2)
Supplement: Supplementary file 1 — Supplementary materials [file 41419_2025_8340_MOESM1_ESM.docx]

**Supplementary materials**

**
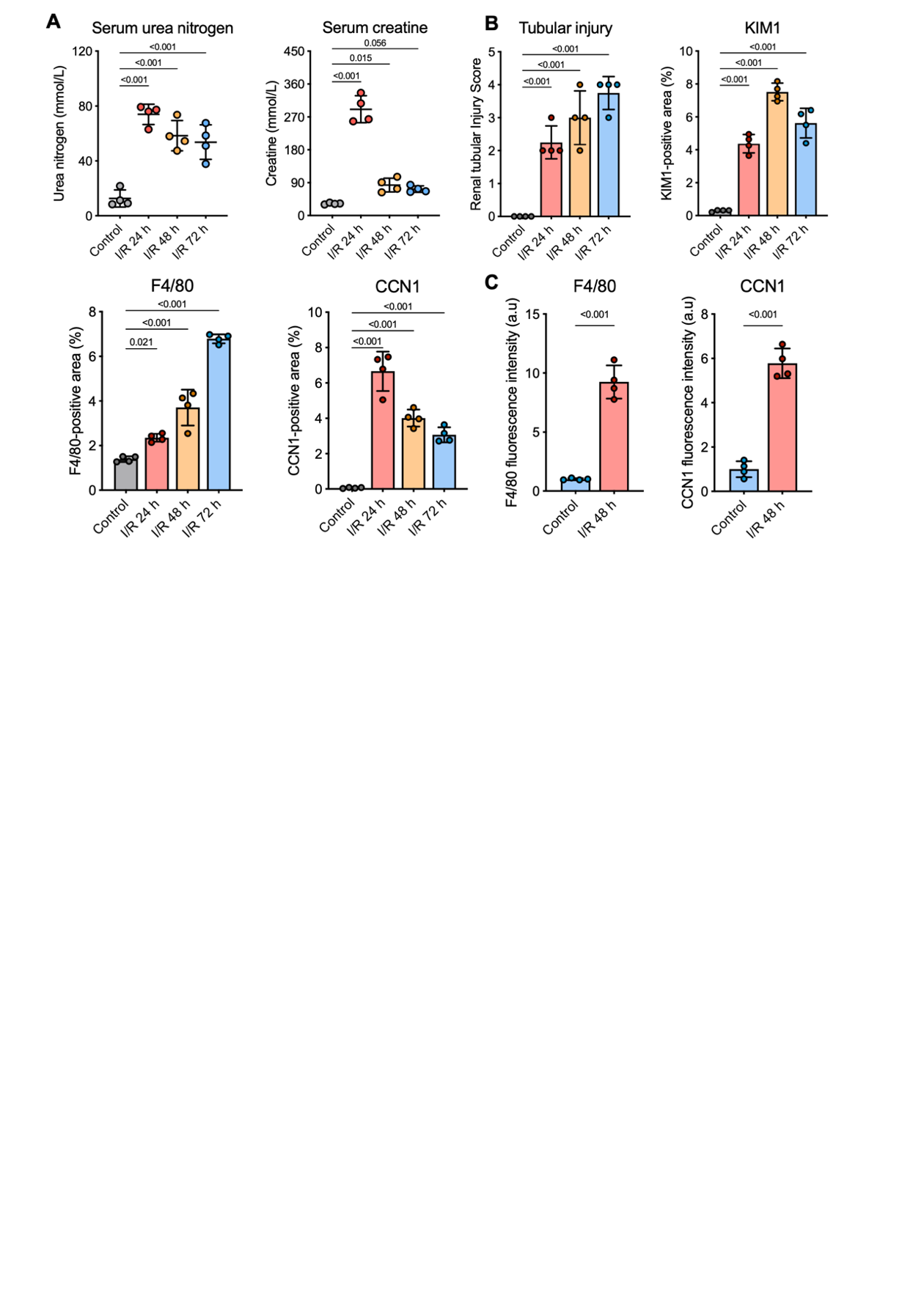
**

**Supplementary Figure 1. CCN1 expression, immune cell infiltration, and renal injury following I/R-AKI.** (A) Blood urea nitrogen and serum creatinine levels in mice after I/R. (B) Quantification of tubular injury scores and positive areas of KIM1, F4/80, and CCN1. (C) Quantification of fluorescence intensity of F4/80, and CCN1. n = 4 mice per group. Data are presented as mean ± SD.


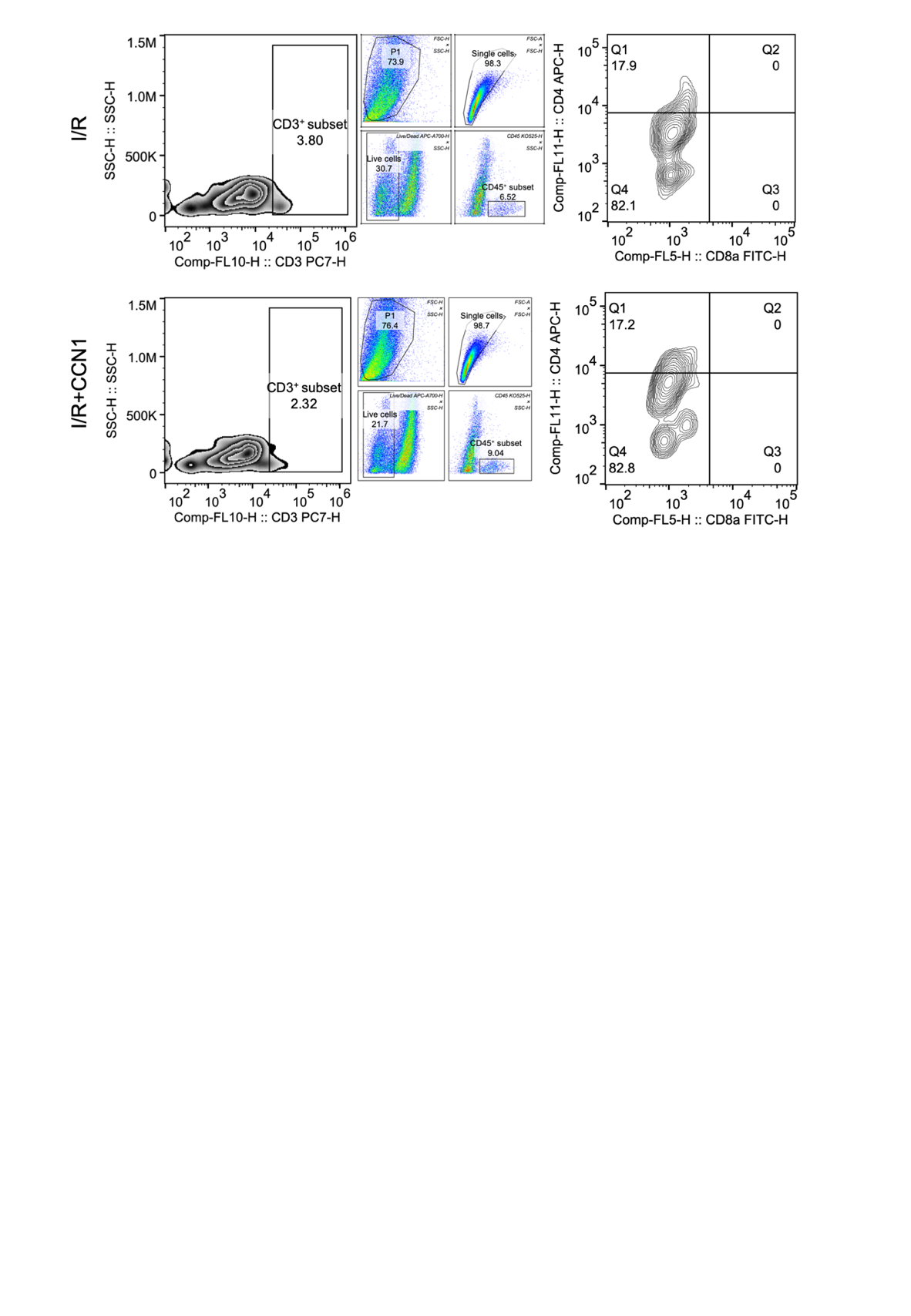


**Supplementary Figure 2. Flow cytometry analysis of renal T cells after I/R.** Quantification of total CD3⁺ T cells and CD4⁺/CD8⁺ subsets in mouse kidneys following I/R.


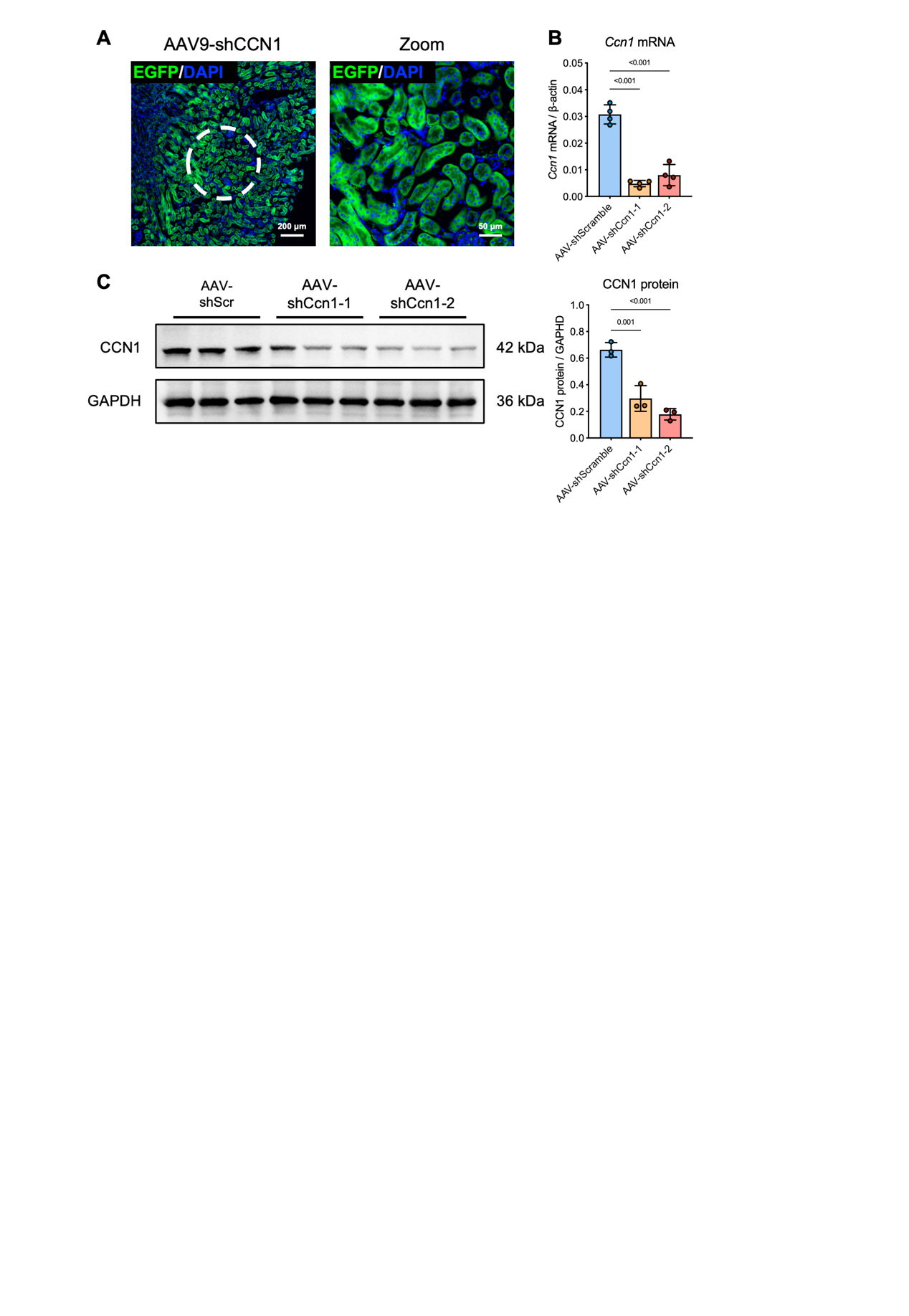


**Supplementary Figure 3. Validation of RTEC-specific CCN1 knockdown using AAV-mediated gene delivery.** (A) EGFP fluorescence of renal tissue demonstrates successful AAV-mediated expression. (B-C) Validation of RTEC-specific CCN1 knockdown by mRNA and protein levels in renal cortex. n = 4 mice per group. Data are presented as mean ± SD.


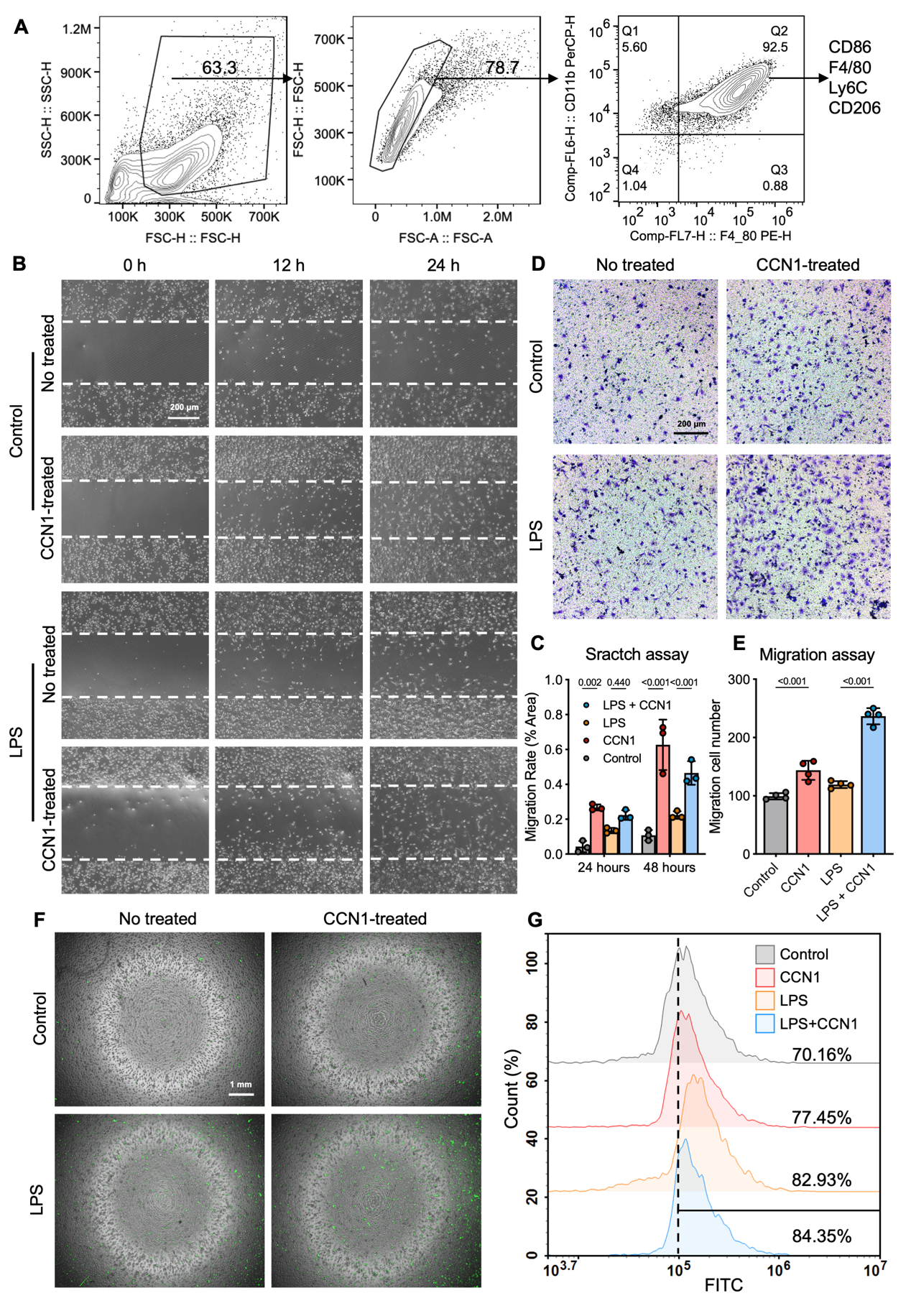


**Supplementary** **Figure 4. Effect of CCN1 on migration and phagocytosis of macrophage**. (A) Gating strategy for CD11b^+^F4/80^+^ BMDMs by flow cytometry analysis. (B-C) Representative images of BMDMs at 0 h, 12 h and 24 h of scratch assay and quantitative analysis of migration area. (D-E) Representative images of BMDMs of transwell migration assay and quantitative analysis of migration cell number. (F) Representative fluorescence image of BMDMs phagocytosing latex beads. (G) Flow cytometry analysis of the proportion of positive cells phagocytosing latex beads in BMDMs. n = 3 per group. Data are presented as mean ± SD.

**
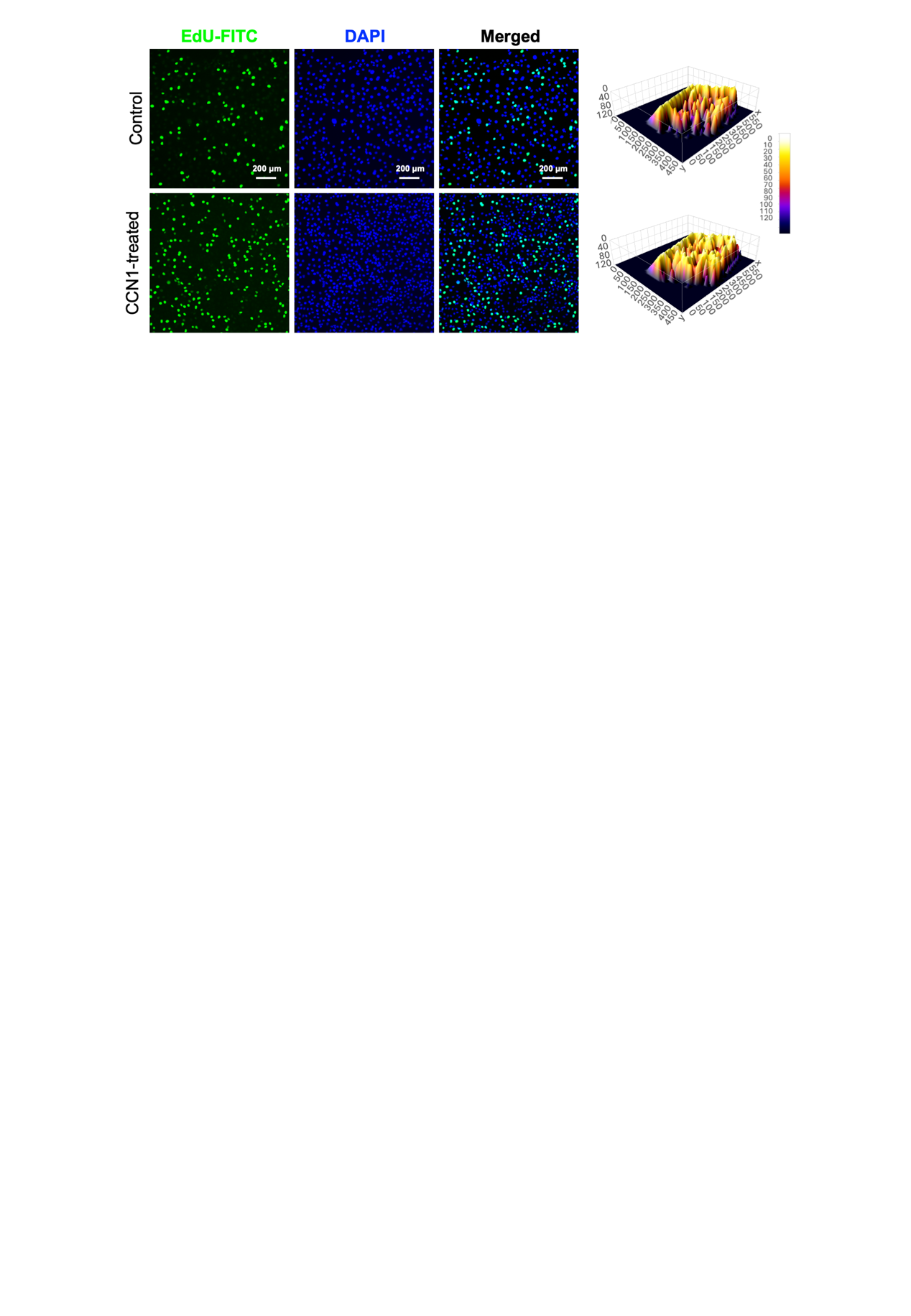
**

**Supplementary Figure 5. CCN1 enhances proliferation of HK-2 cells *in vitro*.** Representative image of EdU immunofluorescence staining and quantification of EdU^+^ proportion in HK-2 cells with CCN1 protein treatment.

**
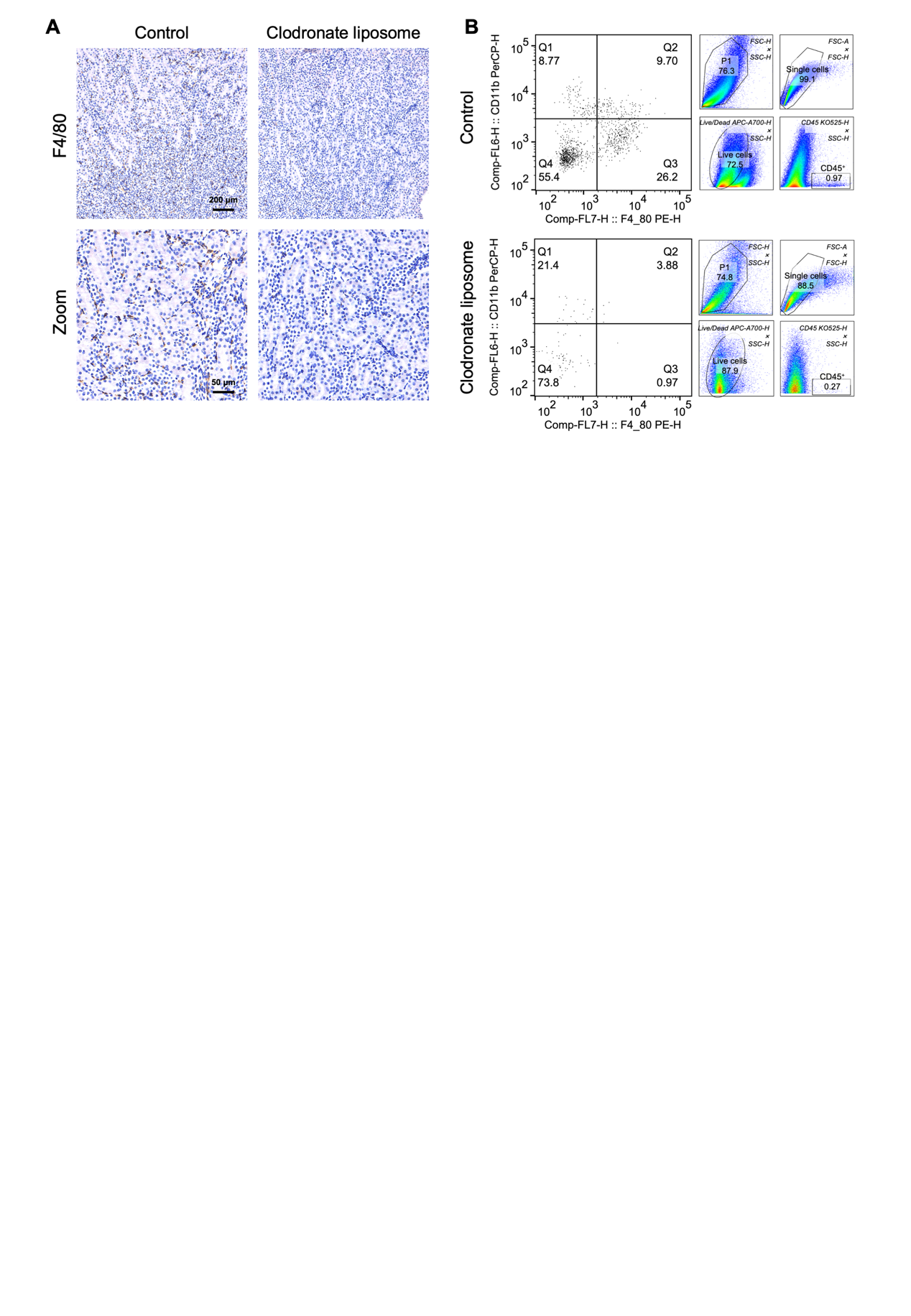
**

**Supplementary** **Figure 6. Construction and validation of a macrophage-depleted mouse model.** (A) Representative images of F4/80 immunostaining in kidney tissues. (B) Representative images of F4/80 immunostaining in kidney tissues.


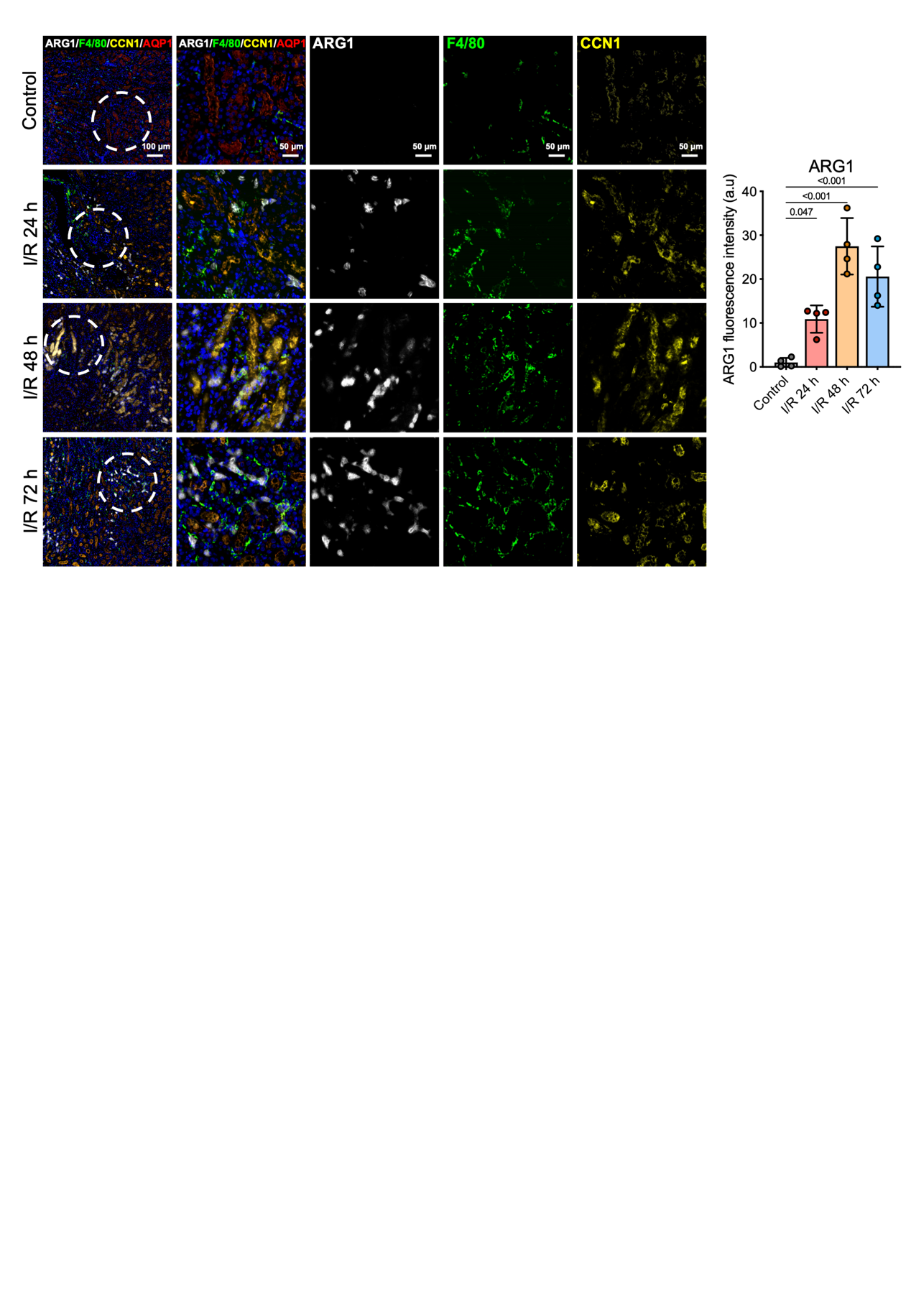


**Supplementary** **Figure 7. Representative fluorescent micrographs and quantification of ARG1, F4/80, CCN1, and AQP1 in I/R-AKI kidneys.** Fluorescent staining of ARG1 (white), F4/80 (green), CCN1 (yellow), and AQP1 (red) in kidneys from I/R-AKI mice. n = 4 mice per group. Data are presented as mean ± SD.

**Supplementary Table 1**

| **Cluster** | **Annotation** |
| --- | --- |
| C1 | *MHC-II*^hi^ KRM, |
| C2 | *Ccl4*^hi^ KRM |
| C3 | *Mrc1*^hi^ KRM |
| C4 | *Slc40a1*^hi^ KRM |
| C5 | *Arg1*^hi^ M𝜑 |
| C6 | *S100a9h*^i^ *Ly6c*^hi^ IM |
| C7 | *Chil3*^hi^ *Ly6c*^hi^I M |
| C8 | *Fn1*^hi^ *Ly6c^hi^* IM |
| C9 | *Hbb-bsh*^i^ *Ly6c*^hi^ IM |
| C10 | *Нр*^hi^ M𝜑 |
| C11 | *Plac8*^hi^ *Ly6c*^lo^ IM |
| C12 | *Ace*^hi^ *Ly6c*^lo^ IM |
| C13 | *MHC-II*^hi^ KRM-like |
| C14 | *IFN*^hi^ M𝜑 |
| C15 | *Арое*^hi^ M𝜑 |
| C16 | Prolif. M𝜑 -1 |
| C17 | Prolif. M𝜑 -2 |
| C18 | Prolif. M𝜑 -3 |
| C19 | Prolif. NK |
| C20 | *mt-Co3*^hi^ M𝜑 |
| C21 | *mt-Co2*^hi^ M𝜑 |
| C22 | *Rpl*^hi^ M𝜑 |

**Supplementary Table 2 Primer sequence**

| **Gene** | **Forward Primer** | **Reverse Primer** |
| --- | --- | --- |
| *Ccn1 (mouse)* | GGAGGTGGAGTTAACGAGAAACA | CCAAGACGTGGTCTGAACGA |
| *CCN1 (human)* | GCTCCCTGTTTTTGGAATGGA | CGGCACTCAGGGTTGTCAT |
| *Vegfa (mouse)* | GTCCGATTGAGACCCTGGTG | TTGACCCTTTCCCTTTCCTCG |
| *Hgf (mouse)* | TGGTCCTGAAGGCTCAGACT | CAGGATTGCAGGTCGAGCAA |
| *Igf1 (mouse)* | GCTCTTCAGTTCGTGTGTGGA | GCCTCCTTAGATCACAGCTCC |
| *Hbegf (mouse)* | TCTTCTTGTCATCGTGGGACT | CACGCCCAACTTCACTTTCT |
| *Pdgfb (mouse)* | CATCCGCTCCTTTGATGATCTT | GTGCTCGGGTCATGTTCAAGT |
| *Arg1 (mouse)* | GTGAAGAACCCACGGTCTGT | ATCGGCCTTTTCTTCCTTCCC |
| *β-actin (mouse)* | ACGGCCAGGTCATCACTATTG | AGAGGTCTTTACGGATGTCAACGT |
| *β-actin (human)* | GGGAAATCGTGCGTGACATT | GGAACCGCTCATTGCCAAT |

**Supplementary Table 3 Antibody Information for Flow Cytometry**

| Antibody | Fluorescent Channel | Brand | Catalog |
| --- | --- | --- | --- |
| anti-mouse CD45 Antibody | BV480 | Biolegend | #752417 |
| anti-mouse CD11b Antibody | Percp-Cyanine5.5 | Biolegend | #101205 |
| anti-mouse F4/80 Antibody | RY586 | BD Pharmingen | #753430 |
| anti-mouse CD86 Antibody | Brilliant Violet 605 | Biolegend | #105125 |
| anti-mouse CD192 (CCR2) Antibody | APC-Cyanine7 | Biolegend | # 150641 |
| anti-mouse Ly-6C Antibody | Brilliant Violet 421 | Biolegend | #128031 |
| anti-mouse CD206 Antibody | Alexa 647 | BD Pharmingen | #568809 |
| anti-mouse ARG1 Antibody | PE-Cyanine7/FITC | Thermo/ Cell Signaling Technology | #25-3697-80/ #93668 |
| Fixable Viability Stain 700 | FVS700 | BD Pharmingen | 564997 |
| anti-mouse CD3 Antibody | PE-Cyanine7 | Biolegend | 100219 |
| anti-mouse CD4 Antibody | APC | STARTER | S0B5239 |
| anti-mouse CD8α Antibody | FITC | STARTER | S0B1534 |
| anti-mouse I-A/I-E | Brilliant Violet 421 | Biolegend | 107632 |
