# Supplementary material for: Tubule-derived CCN1 drives renal repair via αvβ5-STAT6-ARG1-dependent reprogramming of macrophages
Source: Cell Death Dis. 2025 Dec 21;17(1):115. doi: 10.1038/s41419-025-08340-2 (PMC12847786; doi:10.1038/s41419-025-08340-2)
Supplement: Supplementary file 2 — Original Western blots [file 41419_2025_8340_MOESM2_ESM.docx]

Figure 1C


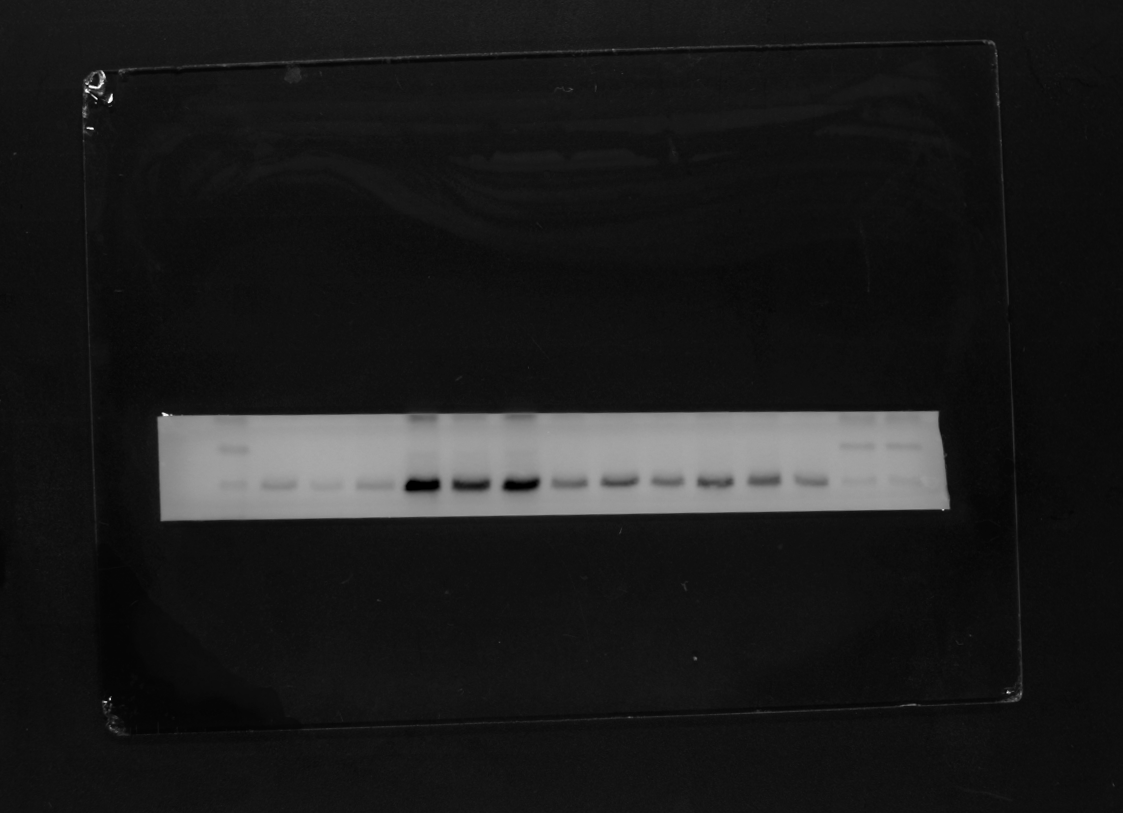


70 KD

55 KD

40 KD CCN1


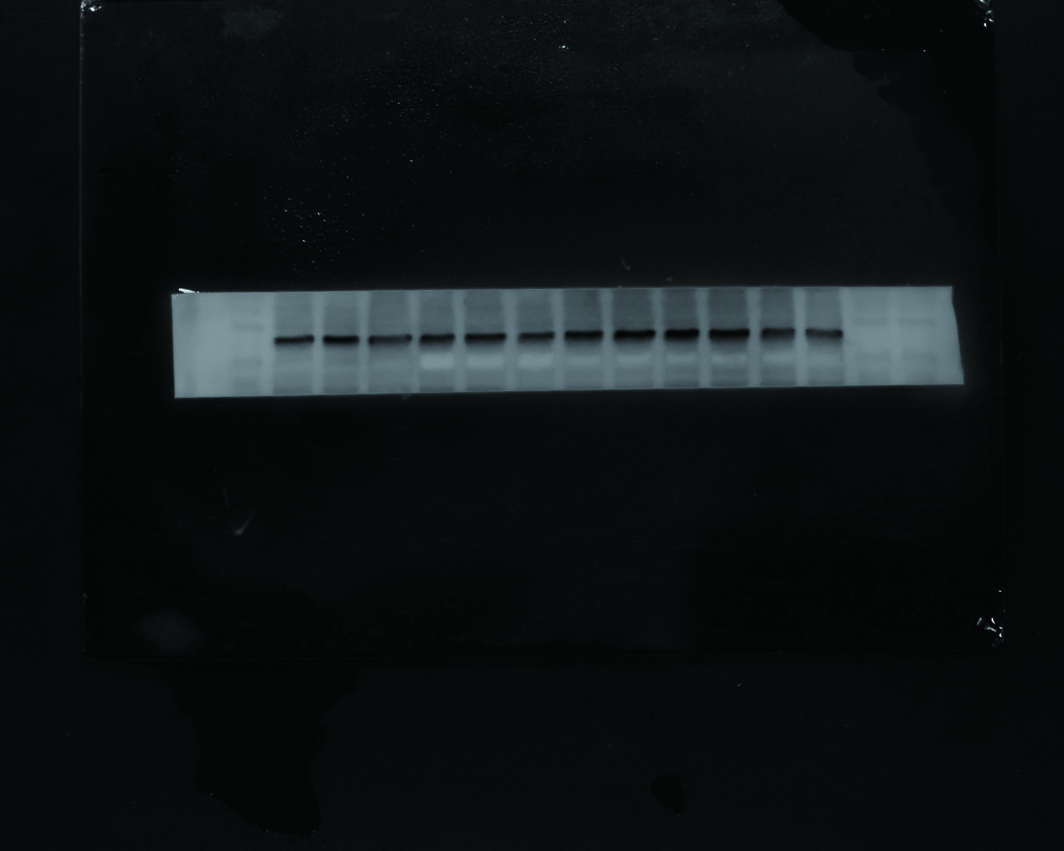


70 KD

55 KD GAPDH

40 KD

Figure 1H


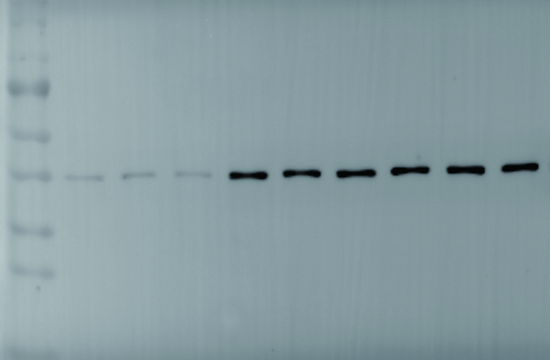


70 KD

55 KD

40 KD CCN1

35 KD

25 KD


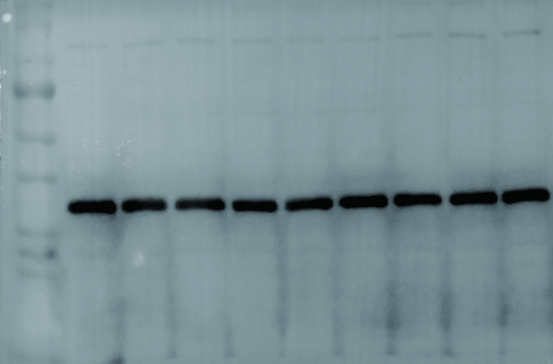


70 KD

55 KD

40 KD

GAPDH

35 KD

25 KD

**Figure 6D**


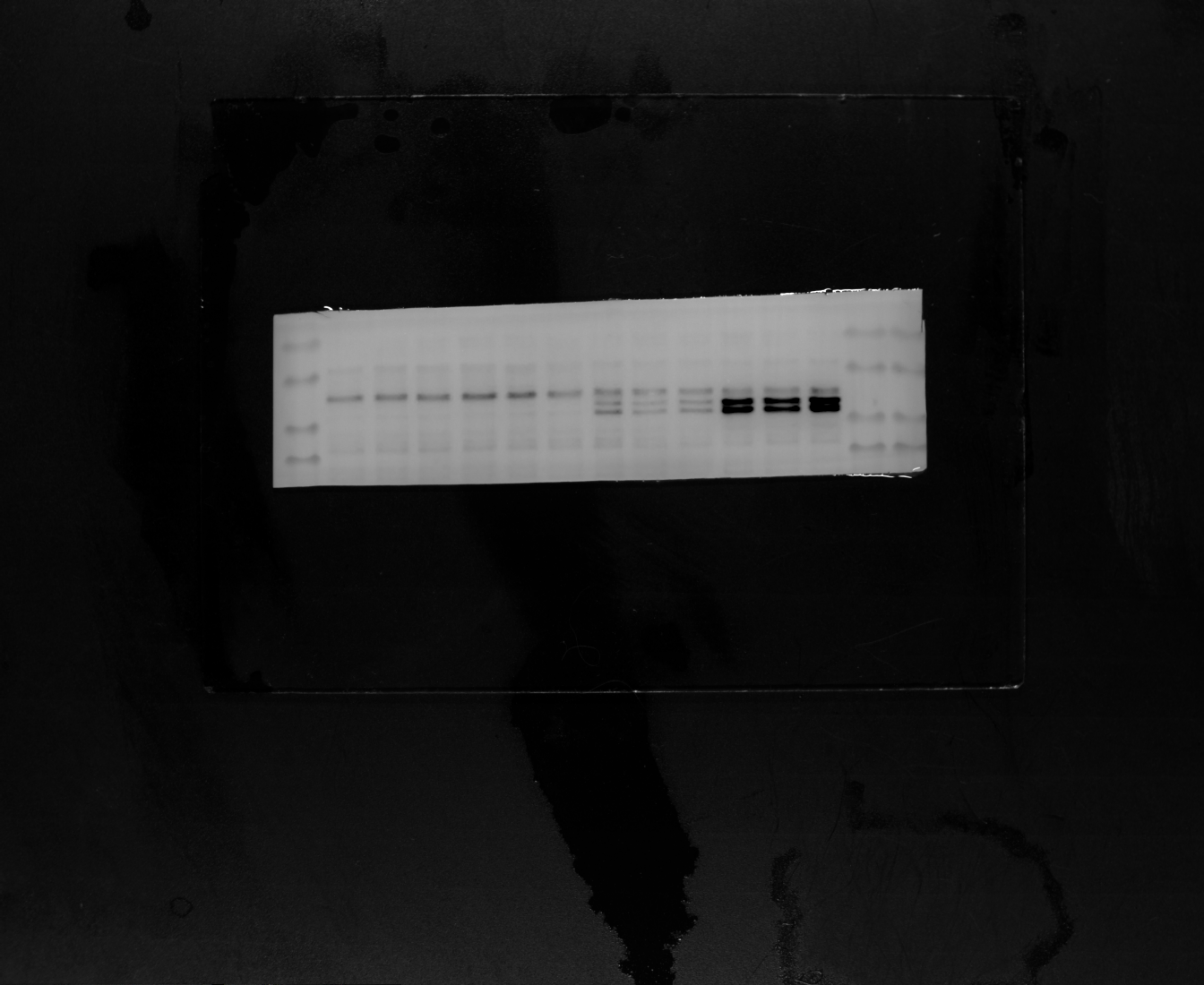


55 KD

40 KD

ARG1

35 KD

25 KD


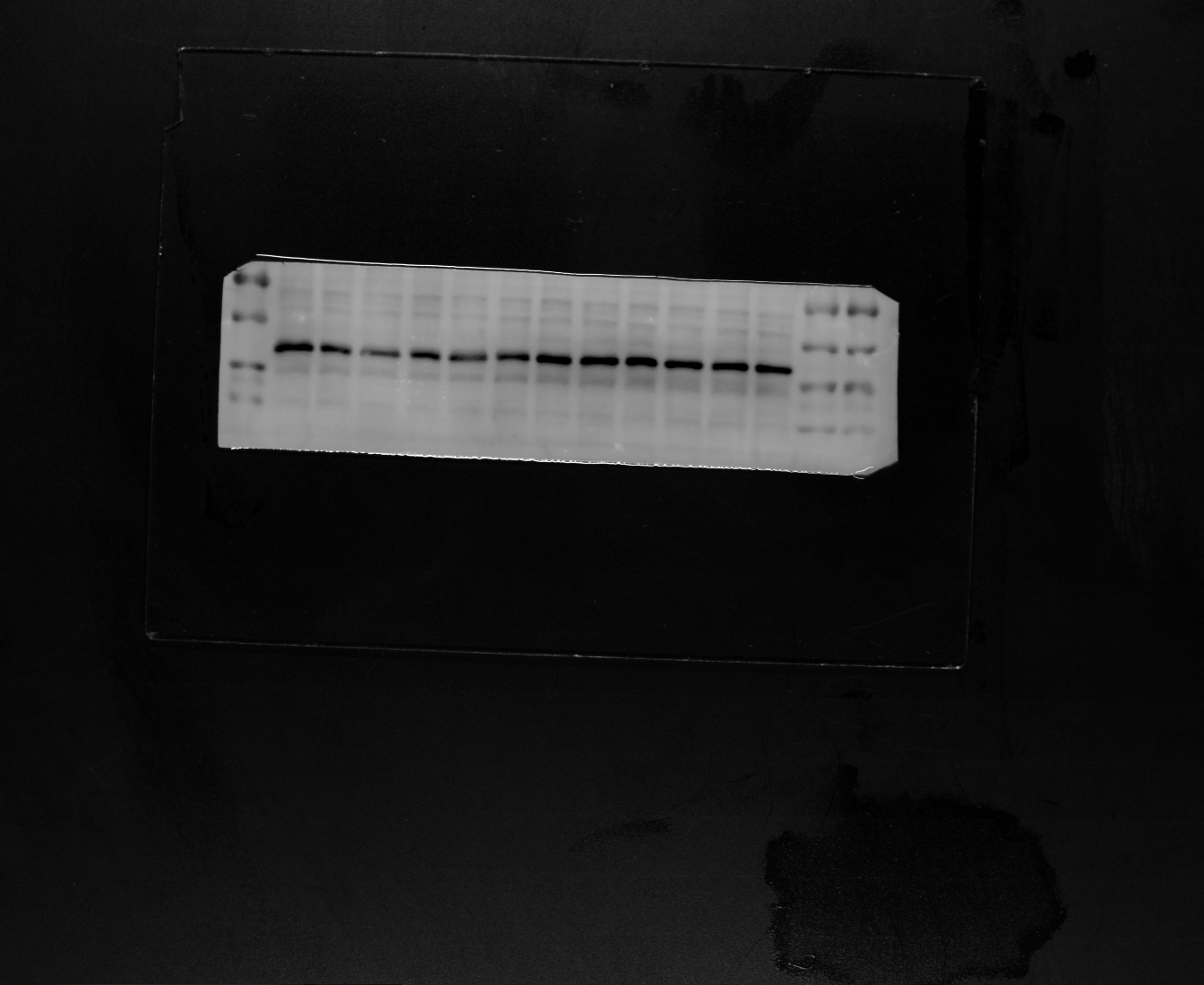


55 KD

40 KD

35 KD GAPDH

25 KD

**Figure 8B**


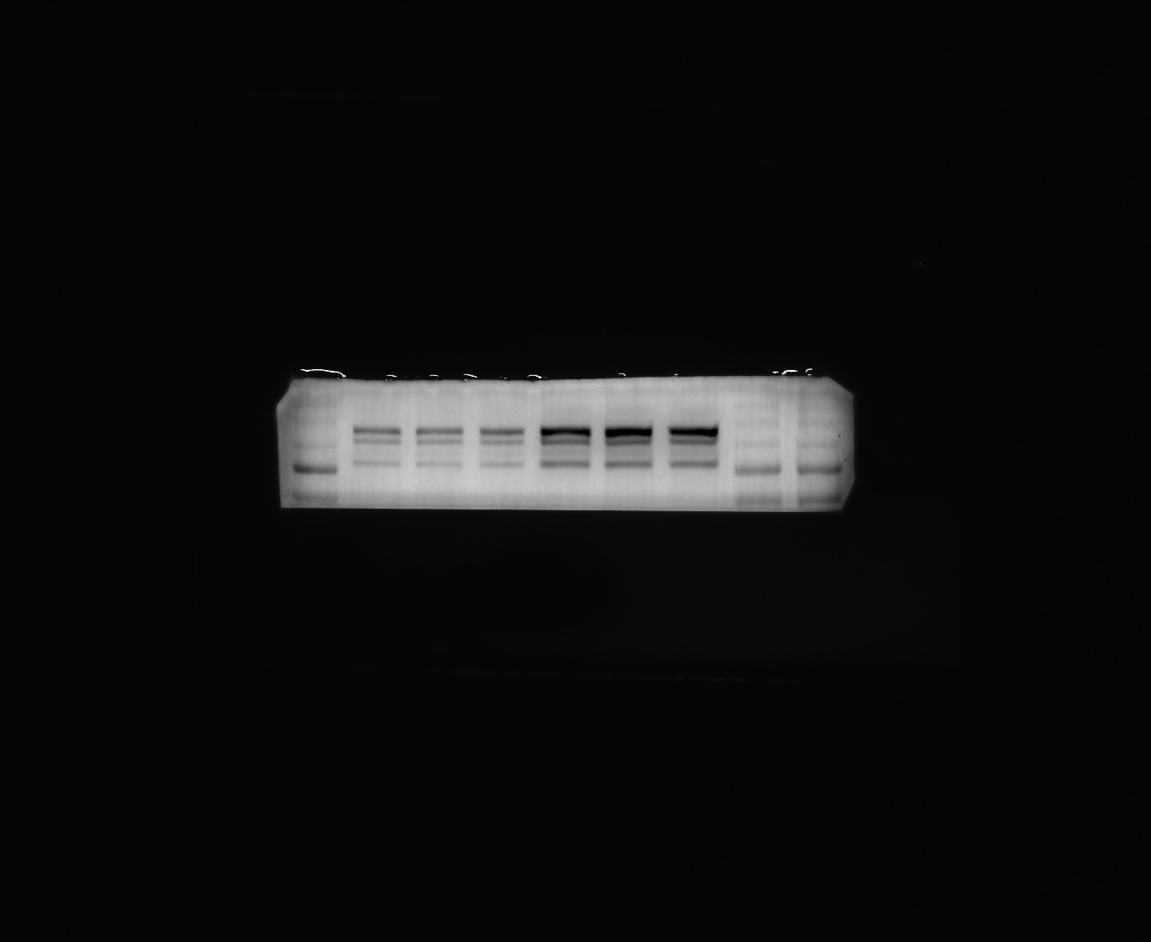


130 KD

100 KD p-STAT6

70 KD

55 KD


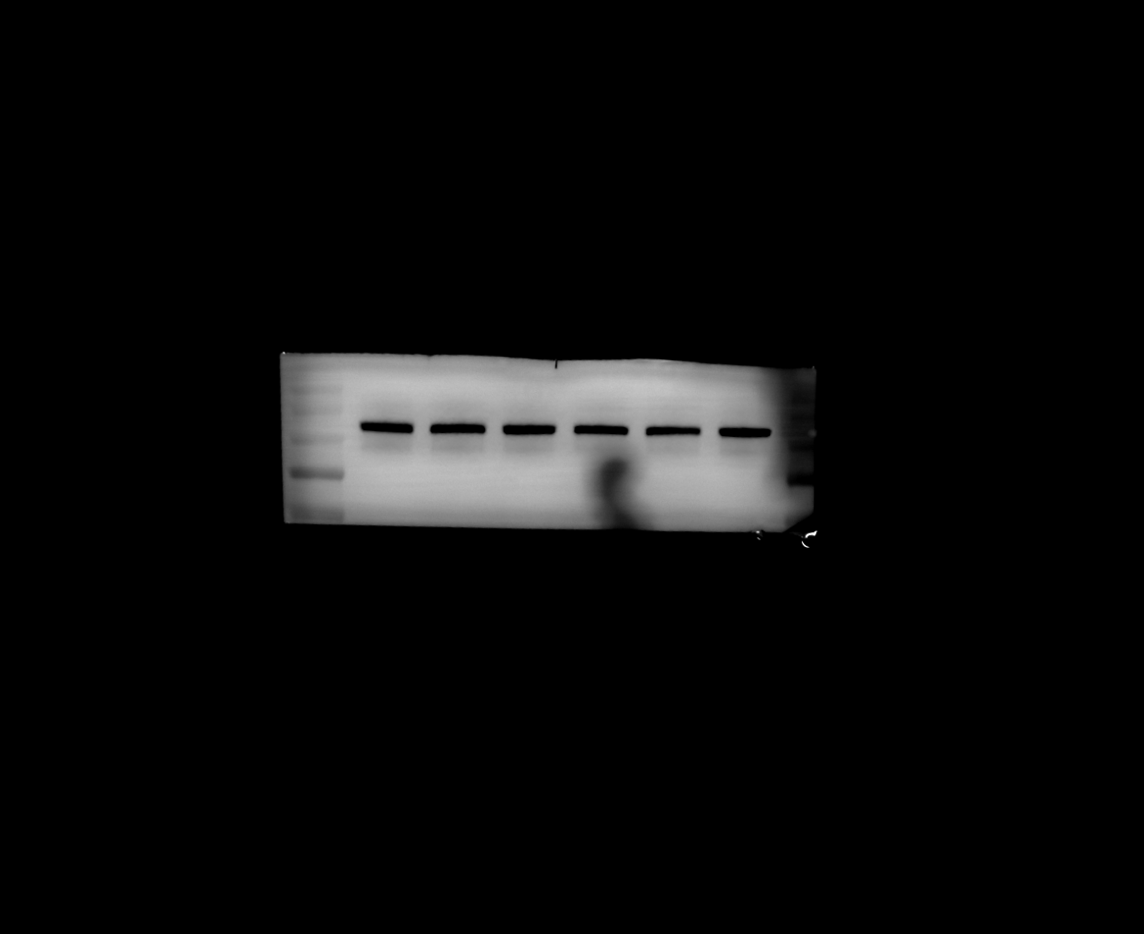


130 KD

100 KD STAT6

70 KD

55 KD


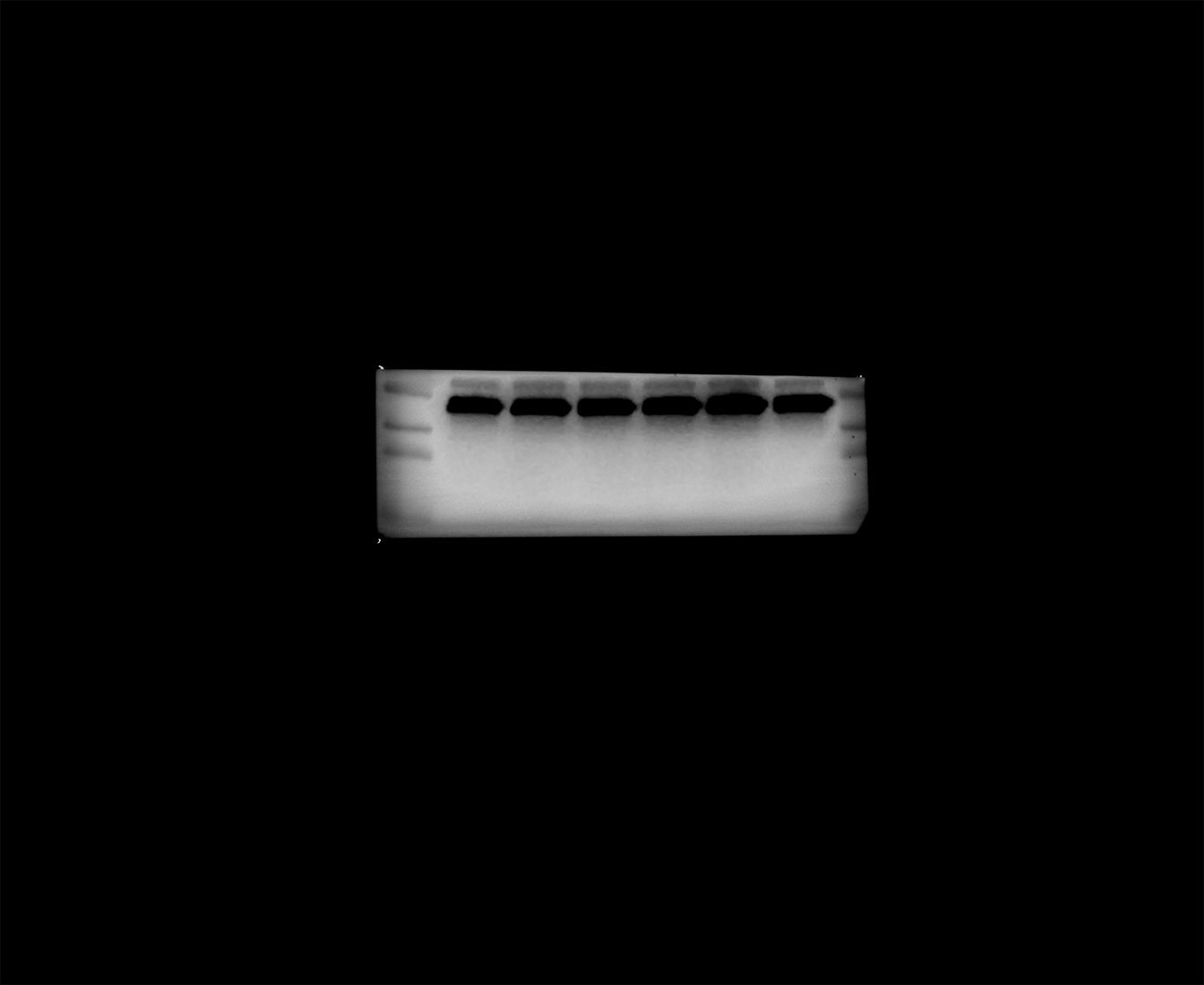


40 KD

GAPDH

35 KD

25 KD


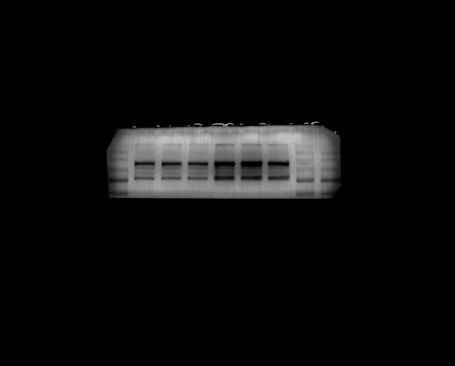


130 KD

100 KD p-STAT6

70 KD

55 KD


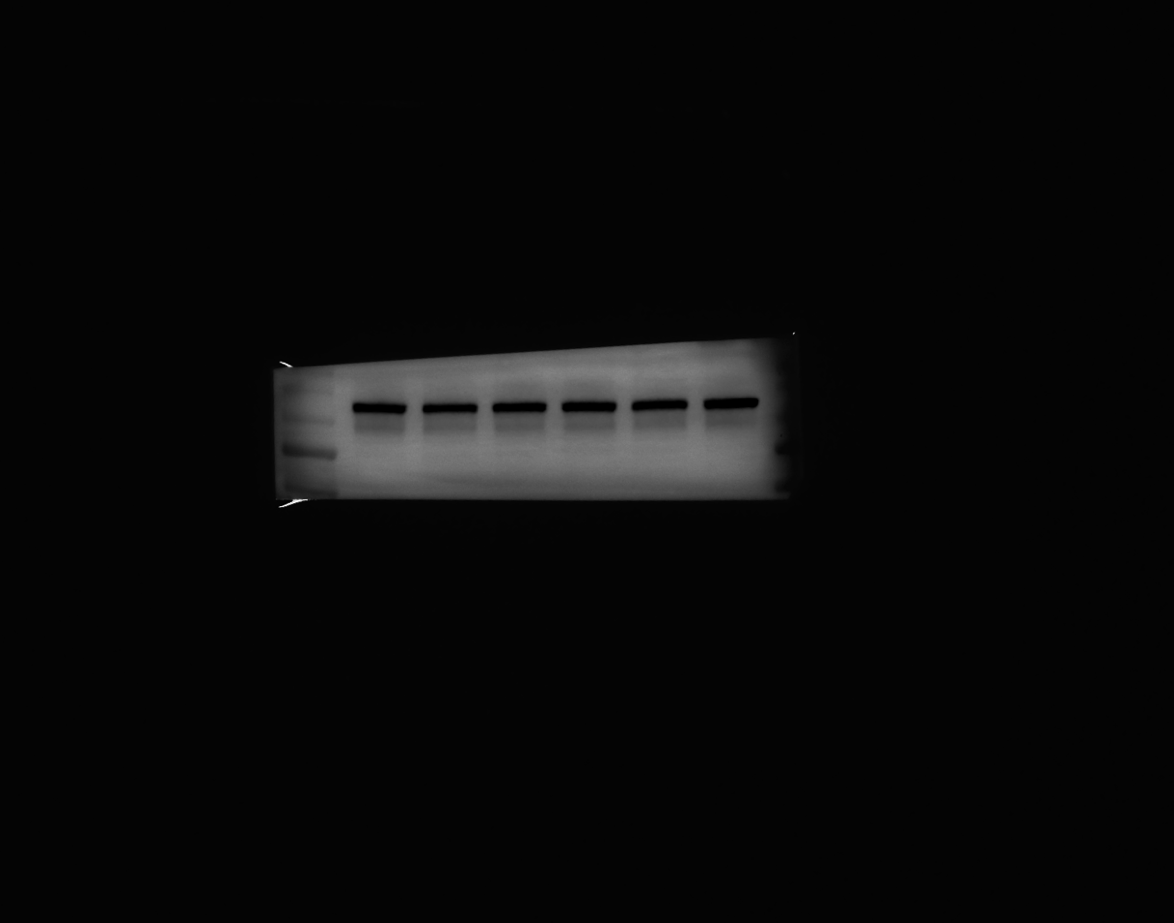


130 KD

100 KD STAT6

70 KD

55 KD


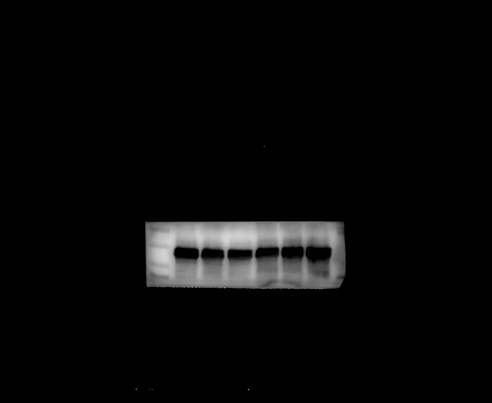


40 KD

40 KD

GAPDH

35 KD

25 KD

**Figure 8F**


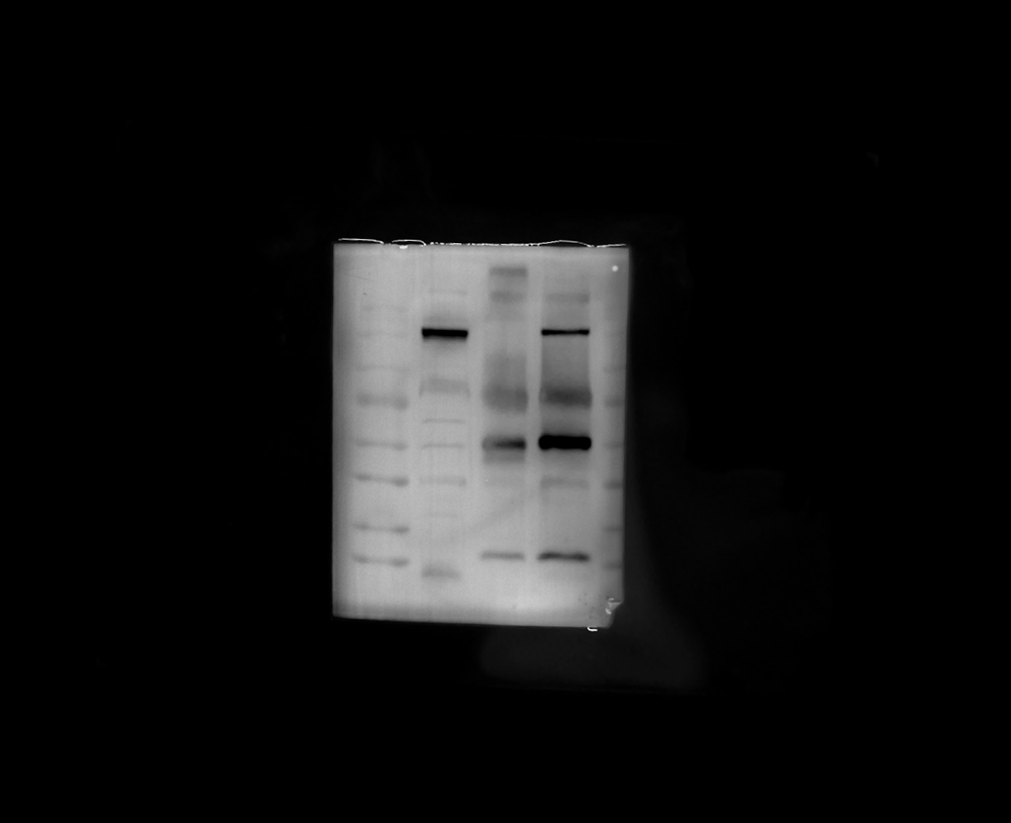
ITGAV

ITGAV

130 KD

100 KD

70 KD Input IgG IP

55 KD

ITGB5


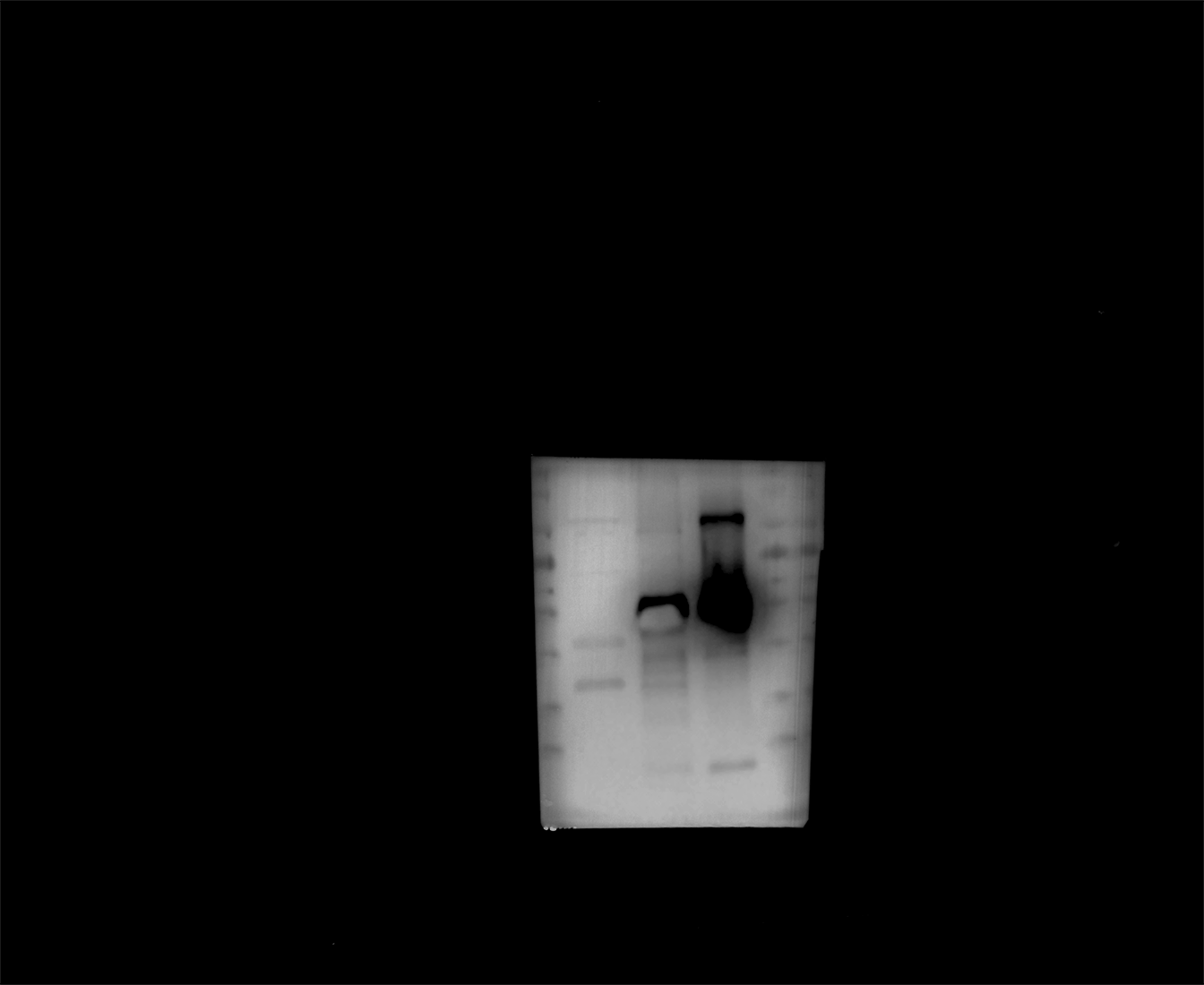

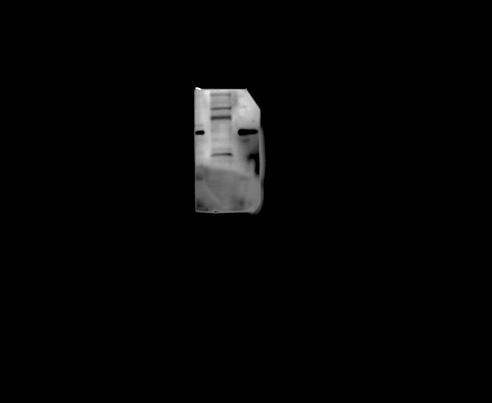


ITGB5 ITGB5

130 KD 130 KD

100 KD 100 KD

70 KD Input 70 KD IgG IP

55 KD 55 KD

CCN1
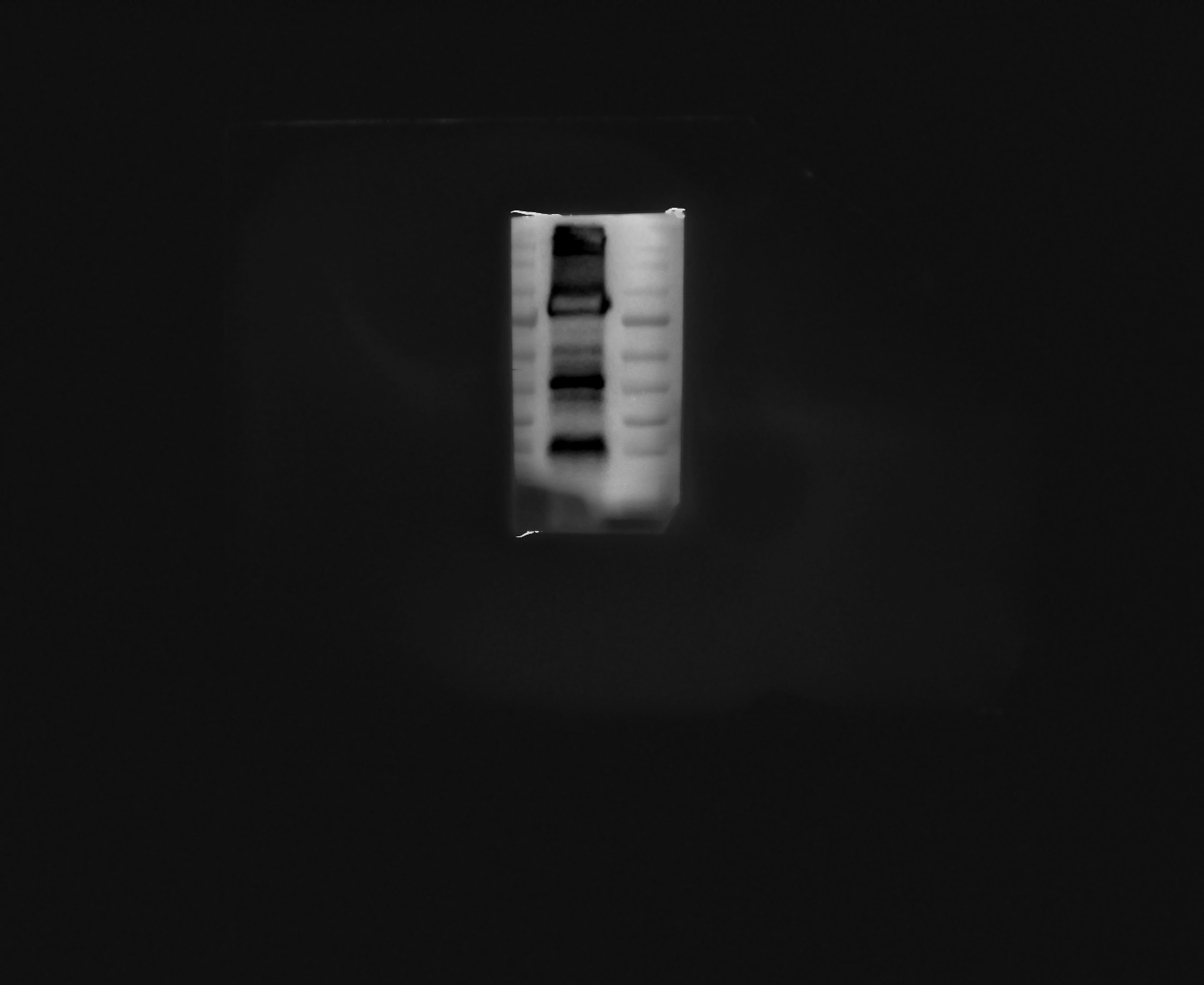


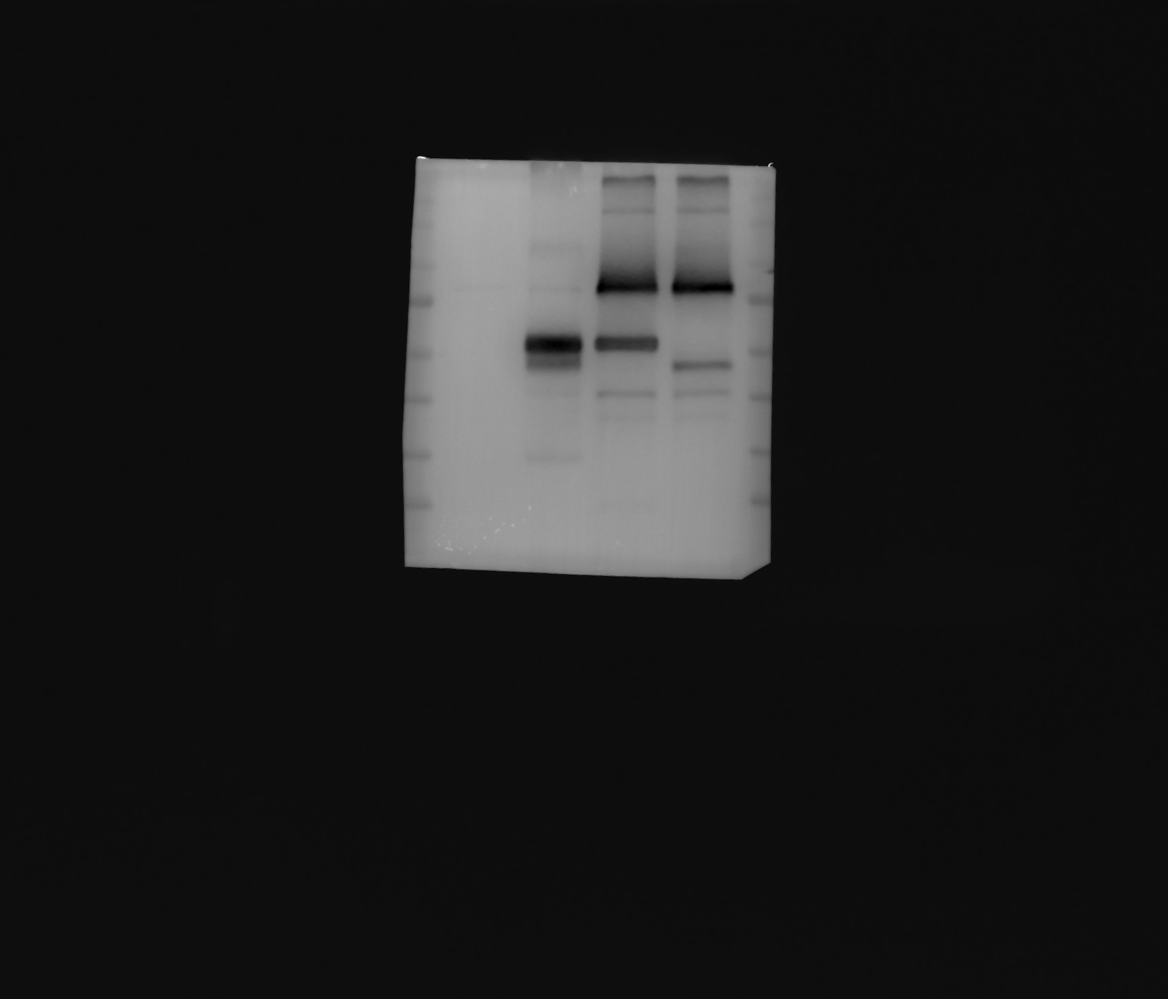


130 KD 130 KD

100 KD 100 KD

70 KD 70 KD

55 KD 55 KD

CCN1 CCN1

Input IgG IP

**Figure 8G**


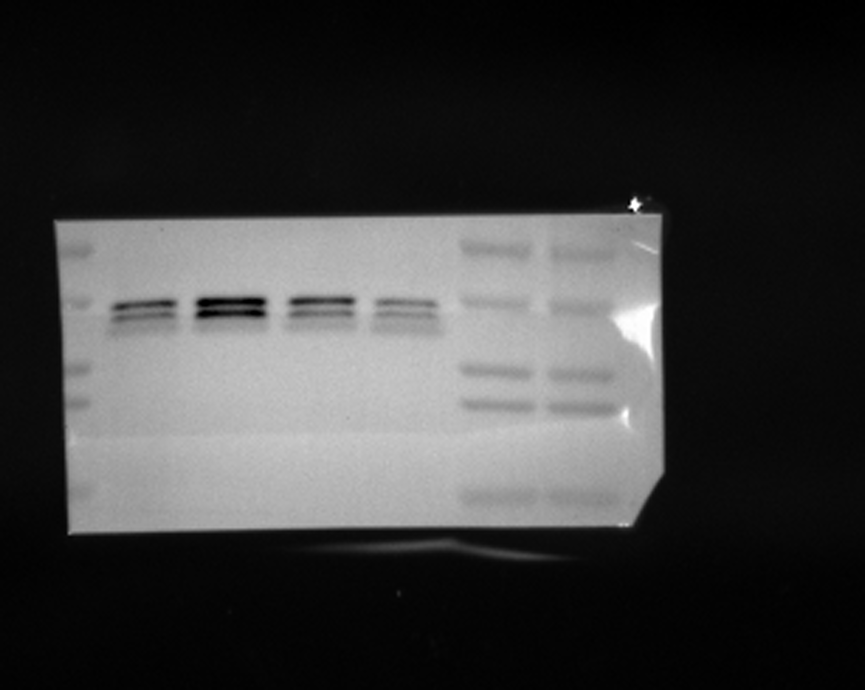


55 KD

40 KD ARG1

35 KD

25 KD


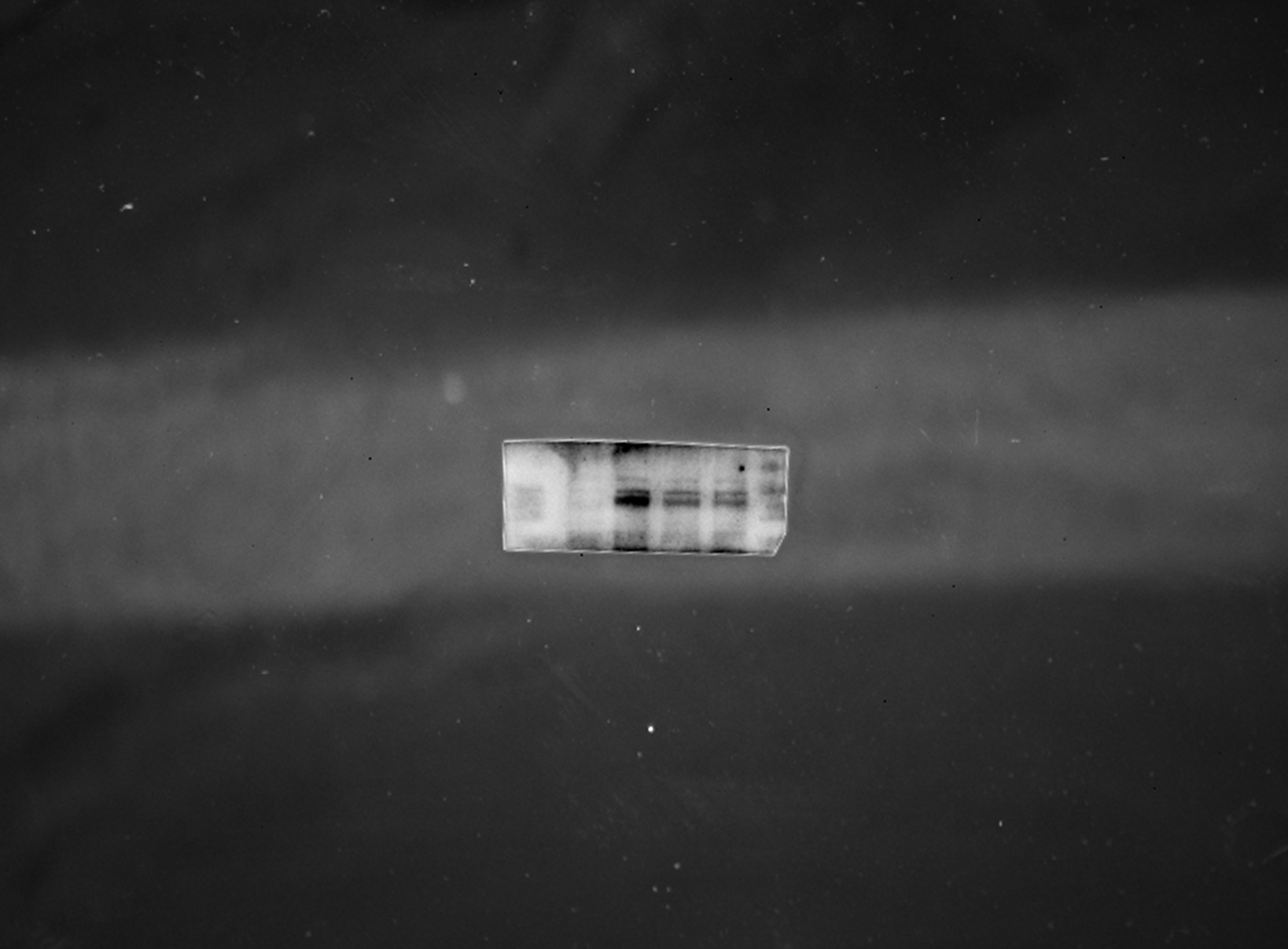


130 KD 130 KD

p-STAT6

100 KD 100 KD

70 KD 70 KD


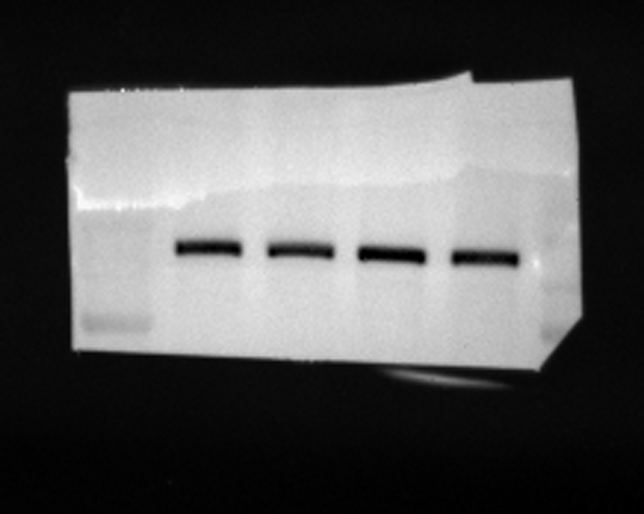


130 KD 130 KD

STAT6

100 KD 100 KD

70 KD 70 KD


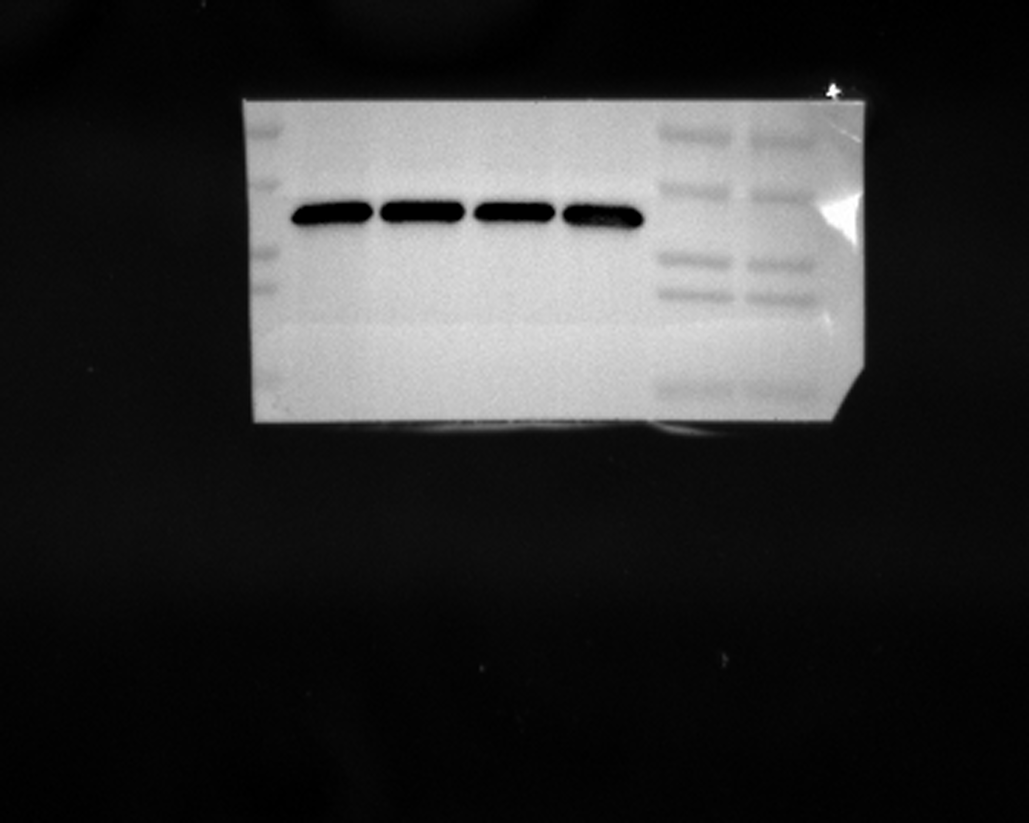


55 KD

40 KD

35 KD GAPDH

25 KD

**Supplementary Figure 3C**


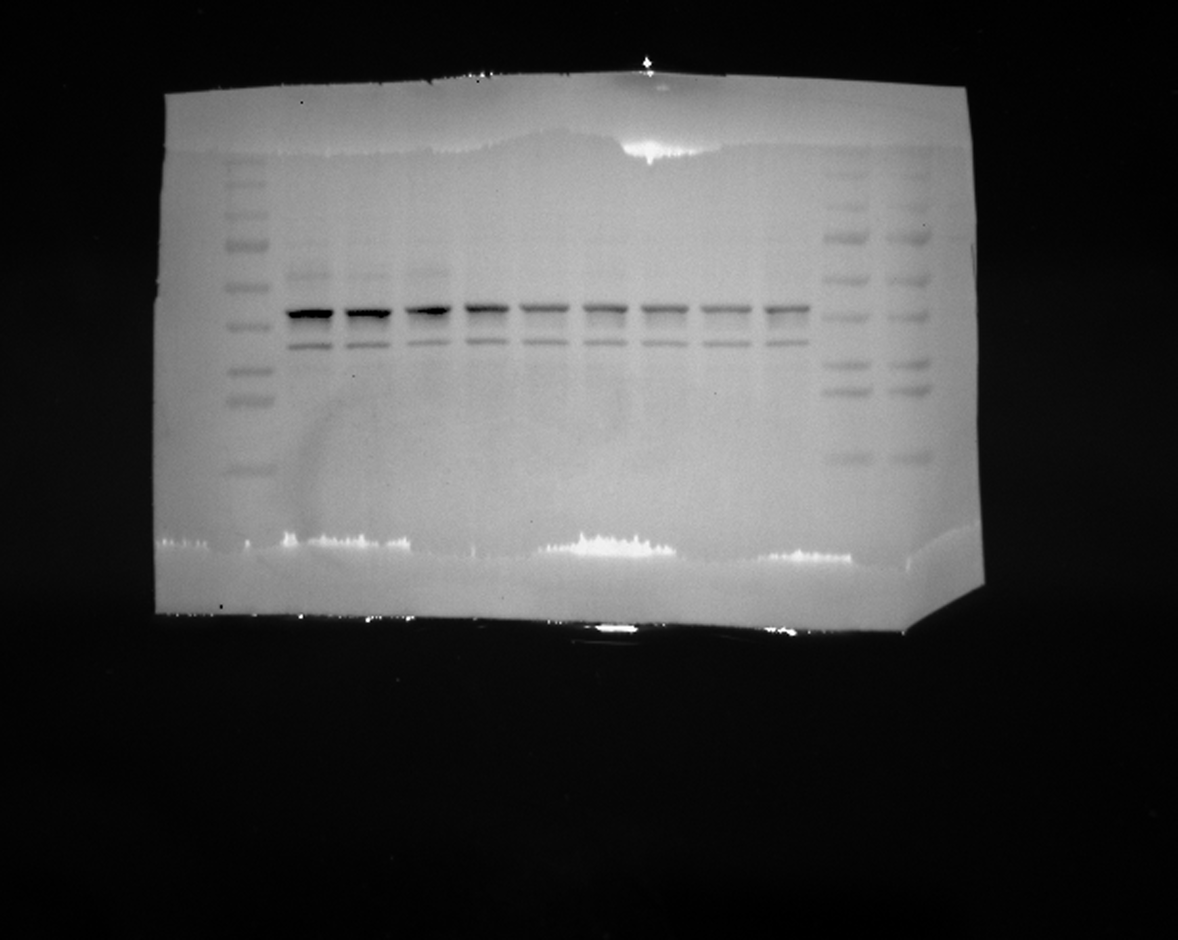


70 KD

55 KD

40 KD CCN1

35 KD


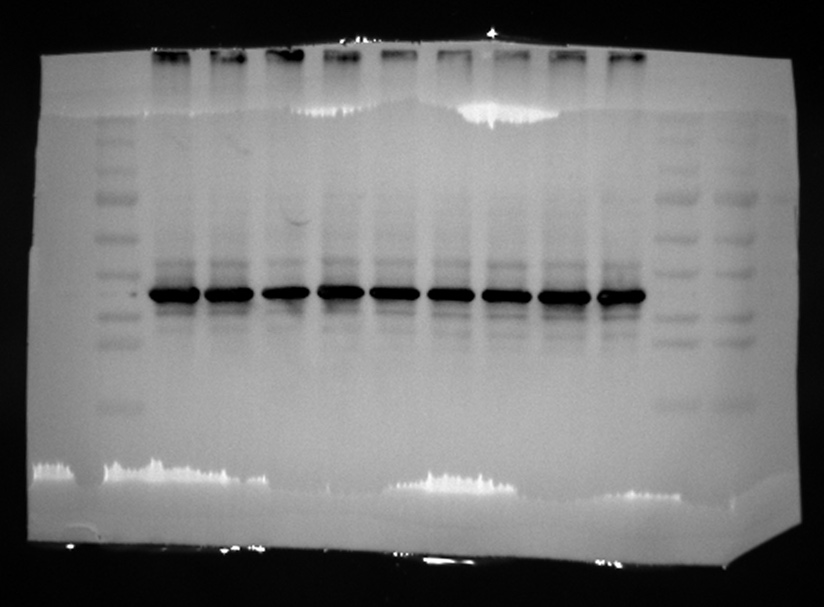


70 KD

55 KD

40 KD GAPDH

35 KD
